# Mechanism of Gu‐Ben‐Hua‐Shi (AESS) Formula Regulating Autophagy Through Hippo‐YAP Pathway in Restoring the Skin Barrier in Atopic Dermatitis

**DOI:** 10.1111/jcmm.71361

**Published:** 2026-09-12

**Authors:** Xin Ma, Yiting Huang, Xiao Lv, Xiumei Mo, Junfeng Liu, Fenggen Yan, Siqi Ye, Yu Zhang, Dacan Chen, Jinjing Jia

**Affiliations:** ^1^ State Key Laboratory of Traditional Chinese Medicine Syndrome/State Key Laboratory of Dampness Syndrome of Chinese Medicine/Department of Dermatology The Second Clinical College of Guangzhou University of Chinese Medicine Guangzhou China

**Keywords:** atopic dermatitis, autophagy, Chinese herbal medicine, skin barrier, traditional Chinese medicine, yes‐associated protein

## Abstract

Atopic dermatitis (AD) is a chronic inflammatory skin disease characterized by skin barrier dysfunction and immune balance disorders. Currently, drugs for repairing the skin barrier in AD are limited. The gu‐ben‐hua‐shi formula (AESS) has been shown to regulate Th1/Th2/Th17/Treg imbalance with good clinical effect. However, its effects on regulating autophagy, repairing the skin barrier, and interacting with Yes‐associated protein (YAP) remain unknown. To elucidate the therapeutic effects of AESS on AD and to investigate its mechanism of restoring skin barrier function through the Hippo‐YAP pathway. We conducted a single‐arm observational study to evaluate the clinical efficacy of the AESS in patients with AD. Network pharmacology was used to predict the potential therapeutic targets and mechanisms. AD mouse models were established by house dust mite stimulation in Nishiki‐nezumi Cinnamon/Nagoya mice, and the effects on autophagy and the skin barrier were measured using western blotting, immunofluorescence, mRFP‐GFP‐LC3 tandem fluorescence assay, and transmission electron microscopy. The effects of AESS serum on an AD‐like inflammatory cell model were detected in HaCaT cells stimulated with interferon‐γ (IFN‐γ)/tumour necrosis factor‐α (TNF‐α). Activity of the Hippo‐YAP signalling pathway was also investigated after YAP knockdown. AESS improved skin rash and barrier function, reduced eosinophil counts, and increased Th1/Th2 and Treg/Th17 ratios in patients with AD. AESS increased autophagy, improved skin barrier function, and inhibited Hippo‐YAP signalling pathway activity. YAP knockdown reduces these therapeutic effects. Besides regulating immune imbalance, AESS increases keratinocyte autophagy levels, thereby restoring skin barrier function in AD through the Hippo‐YAP signalling pathway.

**Trial Registration:** The Chinese clinical trial registration website: ITMCTR202500042

**Abbreviations**
AD: atopic dermatitis
AE: adverse events
AESS: gu‐ben‐hua‐shi formula
DLQI: Dermatology Life Quality Index
EASI: Eczema Area and Severity Index
ELISA: enzyme‐linked immunosorbent assay
FLG: filaggrin
HDM: Dermatophagoides farinae
IFN‐γ: interferon‐γ
IGA: Investigator's Global Assessment
IL: interleukin
IQR: interquartile range
IVL: involucrin
LOR: loricrin
mTOR: mammalian target of rapamycin
NC/Nga: Nishiki‐nezumi Cinnamon/Nagoya
NF‐κB: nuclear factor kappa B
POEM: Patient Oriented Eczema Measure
SCH: stratum corneum hydration
SCORAD: SCORing Atopic Dermatitis
SD: standard deviation
TEWL: trans‐epidermal water loss
Th: T helper cell
TNF‐α: tumour necrosis factor‐α
Treg: regulatory T cells
VAS: Visual Analogue Scale
YAP: yes‐associated protein

## 1 Introduction

Atopic dermatitis (AD) is a chronic, recurrent, inflammatory skin disease that seriously affects quality of life and places a heavy economic burden on patients' families and society as a whole [1]. The overall prevalence of AD worldwide has been increasing [2]. However, modern medicines cannot cure this disease. Local topical corticosteroids, calcium phosphatase inhibitors, and moisturizers are primarily used in patients with mild to moderate AD. However, recurrence cannot be avoided and greatly affects the quality of life and normal social interactions of patients, causing many psychological problems. Long‐term disease recurrence imposes a significant economic burden. Currently, AD ranks first in the burden of nonfatal skin diseases [3].

Skin barrier dysfunction is a hallmark of AD and an initiating factor in its pathogenesis, rendering the skin susceptible to invasion by allergens and microorganisms, leading to immune‐inflammatory disorders. However, long‐term use of existing therapeutic drugs, especially first‐line topical corticosteroids, can worsen the damage to the skin barrier [4]. Existing drugs for repairing the skin barrier at AD sites are limited.

The role of traditional Chinese medicine (TCM) in the treatment of AD is being increasingly recognized internationally. Many AD guidelines mention traditional Chinese medicine as an alternative method for addressing treatment difficulties [5]. AD belongs to the category of ‘Four Bends Wind’ in traditional Chinese medicine, which has a long history of treating AD. According to the principles of TCM, the interaction between an overactive heart fire or embedded dampness and spleen deficiency is the main pathogenesis of ad [6]. The ‘gu‐ben‐hua‐shi formula’ (AESS) is an established formula for treating AD by Professor Dacan Chen, a national leader in traditional Chinese medicine and a famous traditional Chinese medicine practitioner in Guangdong Province. It is derived from the classic Chinese herbal formula ‘san‐shu formula’, which first recorded in *Reverence for the Origin of Materia Medica* (1767 CE, Qing dynasty in China), explains that it is applicable to spleen invigoration and dampness elimination [7]. AESS consists of eight medicinal herbs, including *Atractylodes macrocephala* Koidz. (Baizhu), *Atractylodes lancea* (Thunb.) DC. (Cangzhu), *Curcuma kwangsiensis* S.G.Lee and C.F.Liang (Ezhu), *Lonicera japonica* Thunb. (Jinyinhua), *Sophora jaubertii* Spach (Huaihua), *Coix lacryma‐jobi* L. var. *mayuen* (Roman.) Stapf (Yiyiren), *Saposhnikovia divaricata* (Turcz.) Schischk. (Fangfeng), and *Rehmannia glutinosa* (Gaertn.) DC. (Shengdihuang) (Table 1). AESS has been used in China for decades to treat various inflammatory skin diseases (patent number: ZL202110958138.2), Further information on AESS, including compound preparation and a list of qualitative compounds in the herbs, is presented in Table S1. Recent studies have reported various effects of herbs and their extracts, including anti‐inflammatory [8], barrier repair [9], and immunomodulatory [10] actions, that play important roles in the treatment of AD [11]. Our previous research demonstrated that its optimal dosage effectively alleviated skin lesions in AD mouse models, regulated Th1/Th2/Th17/Treg balance disorders, improved cell proliferation, inhibited apoptosis, and adhered to THP‐1 cells [12]. Yes‐associated protein (YAP) is involved in regulating the proliferation and differentiation of epidermal keratinocytes, wound healing, and the maintenance of the three‐dimensional structure of the skin [13, 14, 15]. This formula is an established herbal formula for treating AD and has been proven effective in clinical applications for many years. However, TCM has a multi‐target and multi‐pathway systemic modulation effect. Although our study noted a decrease in YAP expression in epidermal keratinocytes in patients with AD and in mouse models, previous research has mainly focused on immune disorders, and the association between keratinocytes and the skin barrier remains unclear. Moreover, no reports are available on single‐arm clinical trials.

**TABLE 1:** The composition of gu‐ben‐hua‐shi (AESS) Formula.

Chinese name	Botanical name	English name	Plant part	Batch code	Proportion (g)
Baizhu	*Atractylodes macrocephala* Koidz.	Atractylodis Macrocephalae Rhizoma	Rhizome	2308004	15
Ezhu	*Curcuma kwangsiensis* S.G.Lee and C.F.Liang	Zedoary	Rhizome	230501	10
Cangzhu	*Atractylodes lancea* (Thunb.) DC.	Swordlike Atractylodes Rhizome	Rhizome	2307002	10
Fangfeng	*Saposhnikovia divaricata* (Turcz.) Schischk.	Divaricate Saposhniovia Root	Root	C12309021	10
Huaihua	*Sophora jaubertii* Spach	Sophorae Flos	Flower	2307001	10
Yiyiren	*Coix lacryma‐jobi* L. var. *mayuen* (Roman.) Stapf	Coicis Semen	Seed	230803411	20
Jinyinhua	*Lonicera japonica* Thunb.	Honeysuckle Flower	Flower	2308001	10
Shengdihuang	*Rehmannia glutinosa* (Gaertn.) DC.	Rehmannia Root	Root	C12305214	10

Therefore, the main objectives of this study were to confirm that AESS plays an important role in simultaneously attenuating skin barrier dysfunction and immune disorders, solving the two main pathogenesis of AD, and exploring potential mechanisms involving the Hippo‐YAP‐mediated autophagy pathway. Given the presence of skin barrier dysfunction and decreased autophagy in AD, this study further explored the relationship between YAP expression, decreased autophagy, and skin barrier dysfunction.

## 2 Materials and Methods

### 2.1 Preparation and Qualitative Analysis of AESS

All raw herbs were provided by Guangdong Provincial Hospital of Traditional Chinese Medicine, China. AESS is a mixture of eight medicinal herbs; its composition is shown in Table 1, and all plant names have been checked with http://mpns.kew.org. Specimens were deposited at the Guangdong Provincial Academy of Chinese Medical Sciences. All herbs from AESS were immersed in 10‐fold distilled water for 30 min, decocted with water for 30 min, decocted a second time for 20 min, mixed, and filtered. The drug was concentrated and adjusted to the desired concentration for animal experiments using rotary evaporation.

According to our previous studies, the chemical analysis of the formulation was confirmed using ultra‐high‐performance liquid chromatography/tandem mass spectrometry (UHPLC–MS/MS) analysis [16]. A total of 91 compounds were detected and 76 components of the AESS formula were identified, including 12 organic acids, 1 nucleoside, 2 amino acids, 25 terpenoids, 1 furaldehyde, 3 glycosides, 6 phenylethanoid glycosides, 4 chromones, 9 flavonoids, 12 diarylheptanoids and 16 other‐type components. We also previously analysed the major ingredients in serum after administration [12]. The raw herbs used in this trial were certified to be from the same batch with the same method and results as before (Table S1).

### 2.2 Study Design and Population

This prospective, single‐arm, exploratory clinical trial was conducted between February 2023 and February 2024 at the Dermatology Clinic of the Guangdong Provincial Hospital of Traditional Chinese Medicine (Guangzhou, China). The study protocol was approved by the Ethics Committee of the Guangdong Provincial Hospital of Traditional Chinese Medicine on December 21, 2022 (Approval No.: YF2022‐315‐01), and registered in the clinical trial registry of the World Health Organization (Approval No.: ITMCTR2025000422). The study specifically targeted AD patients who met the Hanifin‐Rajka [17] diagnostic criteria. All participants were required to provide informed consent and sign an informed consent form. The inclusion criteria were: age 18–65 years old, meeting the diagnostic criteria for AD, and Investigator's Global Assessment (IGA) score of ≥ 2. The exclusion criteria included an abnormal blood system, liver, kidney, heart, and other systems; mental disease, malignant tumour, diabetes, and heart disease; pregnancy and lactation; skin diseases that affected the judgement of skin lesions during the clearance period before baseline; and the use of systemic corticosteroids or immunosuppressants within the past 4 weeks.

### 2.3 Interventions

The patients received AESS decoction orally twice daily for 8 weeks, and topical moisturizers were used and standardized for all patients in accordance with the recommended guidelines during treatment, but other topical medications that could affect the results (including corticosteroids, calcineurin inhibitors, and PDE4 inhibitors) were avoided. The skin barrier function was reported as stratum corneum hydration (SCH) and transepidermal water loss (TEWL) using a skin detector (GPSkin Barrier Light, Korea) to measure skin and non‐skin lesions [18]. The measurement was conducted indoors at 20°C–25°C and a relative humidity of 40%–60% [19], while patients cleaned the target area and acclimatized to room conditions for at least 10 min. The values for TEWL (g/m^2^h) and SCH (a.u.) were reported as the mean of three measurements taken at each time point in the same location.

### 2.4 Clinical Sample Collection and Laboratory Analyses

Blood samples were collected before and after treatment to determine routine blood and IgE levels. After centrifugation to separate the serum, interleukin (IL)‐4 and IL‐17A concentrations were determined using human enzyme‐linked immunosorbent assay (ELISA) kits (Multisciences, China) according to the manufacturer's protocol. Peripheral blood samples were analysed using flow cytometry (Agilent, Santa Clara, CA, USA) to determine the proportion of each cell subpopulation. Following established procedures, Th1/Th2/Th17 and Th17/Treg phenotyping kits (BD Biosciences, USA) were conducted to identify the specific antibodies for human CD4, including interferon (IFN)‐γ, IL‐4, IL‐17A, and Foxp3 for Th1, Th2, Th17 and Treg, respectively.

### 2.5 Outcomes

The primary outcome was AD severity scores such as SCORing Atopic Dermatitis (SCORAD), Eczema Area and Severity Index (EASI), and IGA; skin physiological parameters such as TEWL compared to baseline after treatment; and the proportion of patients whose SCORAD scores improved by at least 50%, 75%, and 90% compared to baseline at week 8 (SCORAD50, SCORAD75, and SCORAD90, respectively) [20]. The secondary outcome included quality of life scores such as the Patient‐Oriented Eczema Measure (POEM), Dermatology Life Quality Index (DLQI), and Visual Analog Scale (VAS) for itching and sleep. The primary safety outcome was adverse events (AEs) and abnormal laboratory results. Additionally, due to the difficulty of collecting clinical samples from patients, we conducted network pharmacology analysis, animal and cell experiments to perform mechanistic studies.

### 2.6 Network Pharmacology Analysis

We selected the top 10 blood components of AESS that qualitative analysis by UHPLC‐MS [12], namely five organic acids, three flavonoids, and two chromogenic ketones, as research subjects based on relative abundance in serum. The structures of the identified compounds were searched in the following databases: PharmMapper (http://www.lilab‐ecust.cn/pharmmapper/) with selection thresholds based on a norm fit threshold of ≥ 0.8, HERB (http://herb.ac.cn/Search/) with a score cutoff of ≥ 0.8, SuperPred (http://prediction.charite.de/) with filtering criteria of ≥ 0.7, Swiss Target Prediction (http://www.swisstargetprediction.ch/) databases with a probability threshold value of ≥ 0.2 for target prediction, and TCMSP (https://old.tcmsp‐e.com/tcmsp.php). The keyword ‘atopic dermatitis’ was used to search for relevant targets of AD in the GeneCards (https://www.genecards.org), OMIM (https://omim.org/), DrugBank (https://go.drugbank.com/), DisGeNET (https://www.disgenet.org/), and CTD (http://ctdbase.org/). Genes with a score greater than the median were selected for analysis. All targets were combined and de‐weighted, followed by standardized corrections in the UniProt database. The intersected targets were imported into the STRING database (https://string‐db.org) for protein–protein interaction (PPI) analysis, and the species was designated as ‘ *Homo sapiens* ’ with a confidence score of 0.400. Cytoscape 3.10.3 software was used to construct a ‘compound‐target‐disease’ network and analyse the network topology, with the top 30 targets by degree value being considered as core targets. GO analysis and KEGG pathway enrichment were analysed using R software (version 4.3.1), and the results were statistically visualized at *p* < 0.05 as the filtered criterion.

### 2.7 Animals and Ethical Statement

All experiments were performed in compliance with institutional guidelines for animal care in China and approved by the Experimental Animal Ethics Committee of Guangdong Provincial Hospital of Traditional Chinese Medicine on September 22, 2022 (Approval No.: 2022097). NC/Nga mice (6–8 weeks old) were divided into six groups (three males and three females). The back and both ears were shaved with hair removal cream, and 200 μL of 4% SDS was applied to the shaved area after 24 h. Next, 100 mg of house dust mite (HDM) ointment (Biostir, Osaka, Japan) was added after 2 h. This was applied twice a week for 3 weeks [21, 22]. An equal volume of Vaseline matrix was applied externally to the control group. The YAP silencing group was injected with YAP shRNA lentivirus (Table S2) at the lesion site [23], whereas the standard dose AESS group was treated with 19.53 g/kg [24] AESS by gavage daily from the first day of modelling. The low‐dose AESS group was 1/2 of the standard‐dose AESS group, and the high‐dose AESS group was twice that of the standard‐dose AESS group. The positive control group was treated with 3 mg/kg dexamethasone (Aladdin, China) [25]. The experiment was performed under isoflurane (3% for induction and 1.5% for maintenance) anaesthesia using a TAIJI small‐animal anaesthesia machine (RWD, Shenzhen, China). Euthanasia was performed using an overdose of anaesthesia, in accordance with American Veterinary Medical Association (AVMA) guidelines, and death was confirmed by cessation of respiration and heartbeat, and loss of corneal reflex.

The remaining groups were administered an equal amount of physiological saline by gavage for three consecutive weeks until the end of the experiment on the 21st day, when the mice were euthanized. The scoring method for dermatitis in mice [26] includes four indicators: (1) erythema/haemorrhage, (2) scarring/dryness, (3) oedema, and (4) excoriation/erosion. The severity of each indicator was calculated on a scale of 0–3, where 0 = none, 1 = mild, 2 = moderate, and 3 = severe. The total score represented the dermatitis score of each mouse.

### 2.8 Preparation of Drug Containing Serum of AESS in Rats

Adult male Sprague–Dawley rats weighing 150–200 g were selected and adaptively fed for 1 week before being randomly divided into control and AESS groups. The rats were fasted for 8–12 h before gavage. After 9.76 g/kg [24] AESS formula was administered to rats, and drug‐containing serum was obtained and prepared using the standard procedure as previously described [27].

### 2.9 Cell Culture

The human keratinocyte cell line HaCaT, purchased from Shanghai Xiangf Biotechnology Co. Ltd. (Shanghai, China), was cultured routinely in DMEM medium (Gibco, Waltham, MA, USA) at 37°C and 5% CO_2_. Based on the previous research, there will be cytotoxicity when the concentration of drug containing serum exceeds 20%; thus, the final dosages in our experiments were 5%, 10%, and 15% AESS drug‐containing serum [12, 28]. The positive group was cultured with 20 μg/mL dexamethasone [12]. The YAP knockdown group was transfected with siRNA (Table S2). HaCaT cells were treated for 24 h with a mixture of 10 ng/mL IFN‐γ (SinoBiological, China) and 10 ng/mL tumour necrosis factor (TNF)‐α (SinoBiological) to create an AD‐like inflammatory cell model [29].

### 2.10 Nuclear and Cytoplasmic Fractionation

Nuclear and cytoplasmic fractionation was conducted using the Nuclear and Cytoplasmic Protein Extraction Kit (Wanleibio, China) according to the manufacturer's instructions. Western Blot was then performed to evaluate the subcellular distribution of YAP in both the nucleus and cytoplasm.

### 2.11 Western Blot

Total protein was extracted from skin lesions or cells, and the protein concentration was calibrated using the Bicinchoninic Acid method. Western blot analyses, such as Sodium Dodecyl Sulfate‐Polyacrylamide Gel Electrophoresis, membrane transfer, antibody incubation, and visualization, were used to detect protein expression. The grayscale values of the bands from three independent experiments were calculated using ImageJ software. The primary antibodies used were: YAP (1:1000, Cat #14074; Cell Signalling Technology, USA), p‐YAPS127 (1:1000, Cat #13008; Cell Signalling Technology), Mst1/2 (1:1000, Cat #NBP2‐67624; Novus Biologicals, USA); p‐Mst1/2 (1:1000, Cat #SAB4504042; Sigma Aldrich, USA), Lats1/2 (1:2000, Cat #PA5‐115498; Thermo Fisher, USA), p‐Lats1/2 (1:400, Cat #bs‐7913R; Bioss, USA), filaggrin (FLG, 1:1000, Cat #GTX23137; Gene Tex, Irvine, USA), involucrin (IVL, 1:400, Cat #bs‐23062R; Bioss), loricrin (LOR, 1:1000, Cat #NBP1‐33610; Novus Biologicals), LC3A/B (1:1000, Cat #12741S; Cell Signalling Technology), p62 (1:1000, Cat #39749S; Cell Signalling Technology), mammalian target of rapamycin (mTOR, 1:500, Cat #WL02477; Wanleibio, China), p‐mTOR (1:500, Cat #WL03694; Wanleibio), β‐actin (1:1000, Cat #WL01372; Wanleibio), Histone H3 (1:1000, Cat #WL0984a; Wanleibio).

### 2.12 Enzyme‐Linked Immunosorbent Assay (ELISA)

Peripheral blood was collected from each group of mice and the serum was separated. The serum levels of IgE (Millipore, China), IL‐4 (Millipore), and IL‐17A (Millipore) were determined using the ELISA kits according to the manufacturer's protocol.

### 2.13 Immunofluorescence Double Staining

Double immunofluorescence staining was used to detect the infiltration of CD4+IL‐17A+ double‐positive cells (i.e., Th17 cells) in the dorsal skin lesions of mice in each group and the expression of LC3+ (Cell Signalling Technology) and YAP+ (Santa Cruz Biotechnology, USA) in mice or cells of each group. Cell slides or tissue sections with TritonX‐100 were incubated at room temperature for 30 min. The primary antibody was added and the samples were incubated overnight at 4°C, then the fluorescent secondary antibody was added and the samples were incubated at room temperature for 60 min. After cleaning, DAPI was added to counterstain the nucleus, an anti‐fluorescence quencher was added to seal the slide, and the staining was observed under a fluorescence microscope. The ImageJ software was used to perform a quantitative analysis of fluorescence intensity and colocalisation.

### 2.14 Transmission Electron Microscopy

After fixation, embedding, dehydration, polymerization, ultrathin sectioning, and staining, the formation of autophagosomes (phagosomes, autophagosomes, and autolysosomes) in each group was observed using transmission electron microscopy (Hitachi, Japan).

### 2.15 Cell Proliferation Assay

The proliferative ability of the cells was determined using an MTT assay kit (Wanleibio). Logarithmic growth stage cells were inoculated into a 96 well plate, with 5 × 10^3^ cells per well and five replicates per group. The absorbance of each well was measured at 570 nm using a microplate reader (BIOTEK).

### 2.16 Cell Apoptosis Assay

The cells were inoculated into a 6‐well plate and treated separately according to the experimental group. The cells were digested and collected according to the apoptosis detection kit (Wanleibio); 500 μL Binding Buffer was added to resuspend the cells, 5 μL Annexin V‐FITC was added and mixed well, then 10 μL Propidium Iodide staining solution was added. The cells were incubated at room temperature in the dark for 15 min, and flow cytometry was performed.

### 2.17 Autophagy Flux Assays

Tandem mRFP‐GFP‐LC3 fluorescence analysis was performed to assess the autophagy flux and distinguish between autophagosomes and autolysosomes, following the same steps as before [30]. HaCaT cells were prepared and infected with the mRFP‐GFP‐LC3 adenovirus at a multiplicity of infection (MOI) of 50. After 24 h, proceed with the previously assigned groupings. The fluorescence microscope was then used to observe, photograph, and analyse, and the number of dots per cell = total number of spots/number of cell nuclei in the microscopic field.

### 2.18 Statistical Analysis

Details of sample size calculations are provided in the Supporting Information. Using SPSS 19.0 statistical software analysis, repeated‐measures analysis of variance was used to analyse the intragroup changes in TEWL at baseline and at each treatment point. The means were compared between two groups using a *t*‐test. Comparisons between three or more groups were conducted using analysis of variance followed by appropriate post hoc multiple‐comparison tests; pairwise comparisons between groups were conducted using the LSD method, and Dunnett's T3 method was used for those with uneven variances. *p* < 0.05 indicated a statistically significant difference.

## 3 Results

### 3.1 The Clinical Efficacy and Safety of AESS Against AD

#### 3.1.1 Baseline Characteristics

**FIGURE 1:**
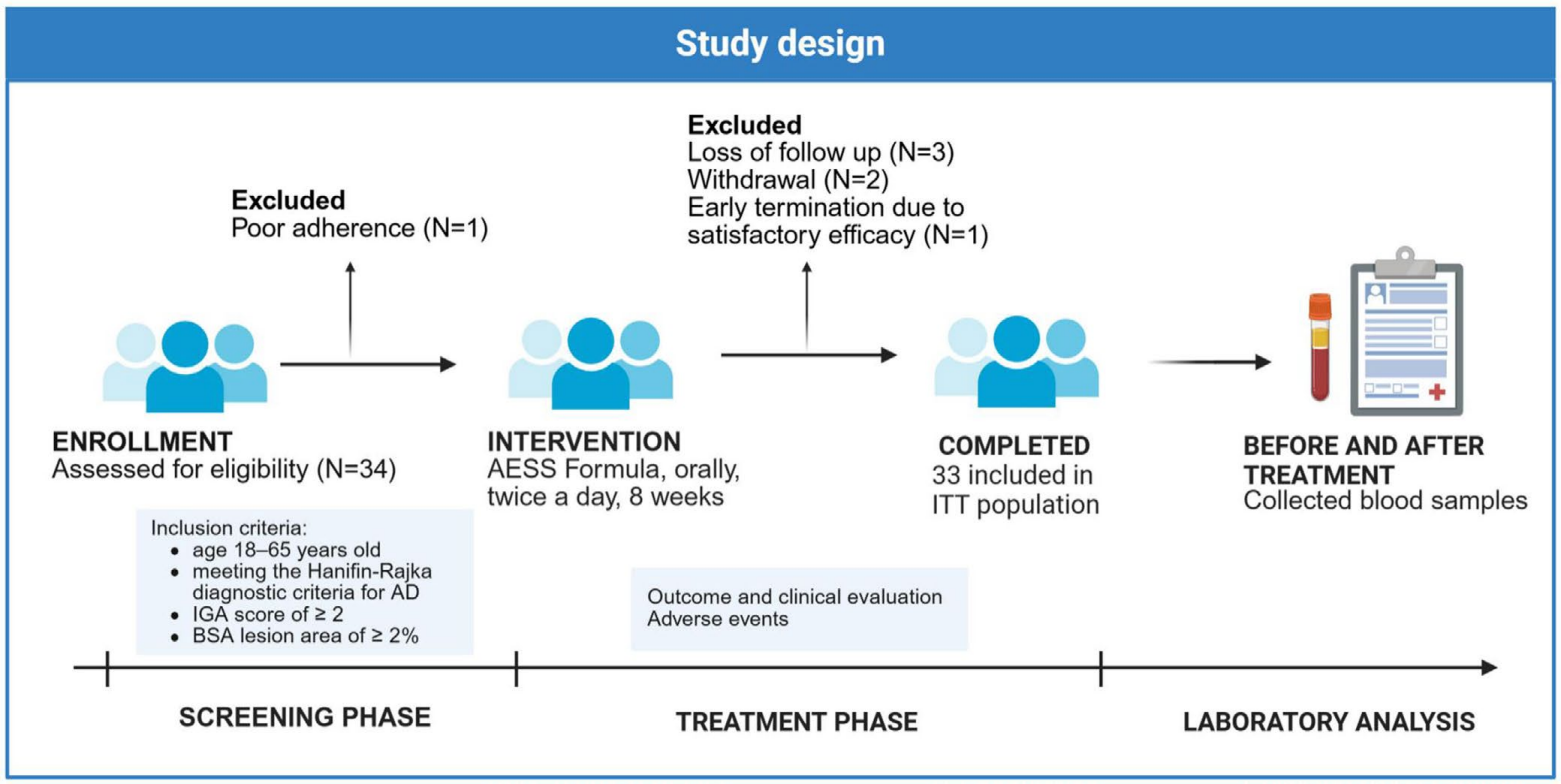
The workflow of the clinical study.

This study included 34 patients, 10 males and 24 females, with an average age of (25.2 ± 6.3) years. One patient was excluded due to poor compliance or failure to complete the trial protocol. Among the 33 remaining participants, 27 completed the entire trial and six dropped out midway. The main reasons for medication discontinuation were patient withdrawal (2/6) and loss to follow‐up (3/6) (Figure 1). The proportion of patients with moderate‐to‐severe AD was relatively high (97%, 33/34), and the IGA score showed that most patients presented with moderate‐to‐severe AD symptoms (74%, 25/34) (Table 2).

**TABLE 2:** Demographics and baseline characteristics.

Characteristics	*N* = 34
Age, median (IQR), years	23.5 (20.8, 30.0)
Sex, *n* (%)	
Male	10 (29.4)
Female	24 (70.6)
Body Mass Index, mean (SD), kg/m^2^	21.0 (3.1)
Disease duration, mean (SD), years	6.4 (7.4)
Baseline IGA, *n* (%)	
IGA 2 (mild)	9 (26.5)
IGA 3 (moderate)	20 (58.8)
IGA 4 (severe)	5 (14.7)
Baseline EASI, mean (SD)	9.3 (6.2)
Baseline SCORAD, mean (SD)	44.9 (11.5)
AD severity, *n* (%)	
Mild	1 (2.9)
Moderate	20 (58.8)
Severe	13 (38.2)
Baseline Pruritus VAS score, mean (SD)	5.3 (2.3)
Baseline Sleepiness VAS score, mean (SD)	3.5 (2.4)
Baseline POEM, mean (SD)	16.7 (6.3)
Baseline DLQI, mean (SD)	12.7 (13.2)
History of allergy, *n* (%)	
Yes	19 (55.9)
No	15 (44.1)

Abbreviations: AD, atopic dermatitis; DLQI, Dermatology Life Quality Index; EASI, Eczema Area and Severity Index; IGA, Investigator's Global Assessment; IQR, interquartile range; POEM, Patient‐Oriented Eczema Measure; SCORAD, SCORing Atopic Dermatitis; SD, standard deviation; VAS, visual analogue scale.

#### 3.1.2 AESS Improves Skin Rash and Reduces TEWL in Patients With AD

Compared with baseline, the main outcome measures, IGA, SCORAD, and EASI scores, significantly decreased over time after 8 weeks of treatment. Secondary outcome measures of the itching index, sleep index, POEM, and DLQI scores were significantly reduced compared to those at baseline. A SCORAD90 response was achieved in eight (24.2%) of the patients in the 8th week, SCORAD75 in thirteen (39.4%), and SCORAD50 in twenty‐five (73.5%). No significant adverse reactions were observed during the study period (Table 3).

**TABLE 3:** Clinical efficacy outcomes from Baseline to Week 8.

Variable	Baseline	End of treatment	*p* (vs. Baseline)
Primary End Point			
IGA, mean (SD)	2.9 (0.7)	0.6 (0.8)	< 0.001***
SCORAD, mean (SD)	44.8 (11.6)	11.6 (12.3)	< 0.001***
EASI, mean (SD)	9.2 (6.7)	1.1 (2.5)	< 0.001***
Secondary End Points			
Pruritus VAS score, mean (SD)	5.0 (2.2)	2.5 (1.7)	< 0.001***
Sleepiness VAS score, mean (SD)	3.4 (2.4)	1.6 (1.6)	< 0.001***
POEM, mean (SD)	16.7 (6.3)	8.3 (6.2)	< 0.001***
DLQI, mean (SD)	13.4 (14.6)	5.4 (4.1)	< 0.001***
Change in Severity Scores, *n* (%)			
SCORAD 90 responder	8 (24.2)		
SCORAD 75 responder	13 (39.4)		
SCORAD 50 responder	25 (73.5)		

Abbreviations: DLQI, Dermatology Life Quality Index; EASI, Eczema Area and Severity Index; IGA, Investigator's Global Assessment; POEM, Patient‐Oriented Eczema Measure; SCORAD, SCORing Atopic Dermatitis; SD, standard deviation; VAS, visual analogue scale.

*** *p* < 0.001.

Compared with baseline, the SCH of non‐lesion areas significantly increased after treatment, and the average SCH score of lesion areas showed an upward trend. At week 8, the TEWL in the lesion area decreased significantly compared to that before treatment, whereas the non‐lesion area showed a decreasing trend. There was no statistically significant difference in TEWL between the lesion and non‐lesion areas; however, the TEWL of the non‐lesion area in the left upper arm decreased over time. Compared to baseline, TEWL in the non‐lesional areas of both upper arms decreased at week 4. Compared to the non‐lesioned areas, the lesioned areas of both forearms, cubital fossa, and upper arms showed statistically significant increases in the second week. Compared to other areas, the TEWL of the bilateral cubital fossa increased over time (Tables 4 and 5).

**TABLE 4:** Change in epidermal barrier function from Baseline to Week 8.

Variable	Area	Baseline	End of treatment	*p* (vs. Baseline)
SCH, mean (SD), a.u.	Lesional	25.5 (17.7)	34.3 (15.5)	0.520
	Nonlesional	30.7 (10.4)	34.9 (10.9)	0.046*
TEWL, mean (SD), g/m^2^ h	Lesional	18.8 (8.0)	12.8 (5.7)	0.040*
	Nonlesional	9.7 (4.2)	9.1 (3.5)	0.443

Abbreviations: SCH, stratum corneum hydration; SD, standard deviation; TEWL, trans‐epidermal water loss.

* *p* < 0.05.

**TABLE 5:** Change in different body regions of TEWL over time (mean (SD), g/m^2^ h).

Variable		Baseline	Week 2	Week 4	Week 6	Week 8	*F*	*p*
Right forearm	Lesional	11.3 (6.4)	18.8 (4.2)**	15.4 (13.5)	19.8 (12.1)	11.5 (5.8)	1.239	0.383
	Nonlesional	7.8 (4.2)	6.9 (4.0)	7.3 (5.5)	7.2 (5.1)	7.3 (3.9)	0.380	0.755
Left forearm	Lesional	13.8 (8.4)	14.2 (6.8)	9.4 (3.9)	11.0 (5.2)	13.3 (9.0)	11.651	0.181
	Nonlesional	7.6 (4.7)	6.5 (3.7)	8.2 (5.1)	7.4 (4.5)	7.9 (4.1)	1.090	0.346
Right cubital fossa	Lesional	20.4 (5.9)	20.3 (9.6)**	17.7 (12.5)	13.0 (5.6)	13.0 (6.0)	2.155	0.238
	Nonlesional	15.2 (8.3)	15.4 (8.0)	12.6 (7.1)	11.8 (7.1)	14.3 (7.0)	1.759	0.174
Left cubital fossa	Lesional	20.9 (9.6)	22.3 (8.9)**	17.4 (10.8)	15.4 (8.3)	16.5 (13.0)	0.475	0.573
	Nonlesional	14.0 (7.9)	15.7 (7.2)*	13.4 (7.7)	13.5 (7.0)***	13.1 (6.6)	0.902	0.442
Right upperarm	Lesional	16.0 (12.4)**	14.4 (10.4)**	11.6 (11.4)	13.1 (10.4)	8.8 (8.0)	2.271	0.373
	Nonlesional	7.6 (3.9)	6.2 (3.7)*	5.9 (4.6)*	6.4 (4.6)	6.8 (4.5)	2.428	0.078
Left upperarm	Lesional	16.3 (10.2)**	13.3 (8.4)**	11.7 (10.2)	10.3 (5.2)**	12.0 (7.6)**	1.018	0.429
	Nonlesional	7.8 (5.2)	6.4 (3.1)	5.3 (4.3)*	5.8 (3.6)*	6.0 (3.7)*	3.538	0.027*

Abbreviations: SD, standard deviation; TEWL, trans‐epidermal water loss.

* *p* < 0.05 versus baseline.

** *p* < 0.05 versus nonlesional areas.

*** *p* < 0.05 comparison between the left and right sides in the same location.

#### 3.1.3 AESS Ameliorates Inflammatory Response in Patients With AD

This study included 34 and 23 patients who underwent serum total immunoglobulin E (IgE) and eosinophil tests before and after treatment, respectively. Compared with baseline, the levels of immunoglobulin IgE showed a decreasing trend after treatment, and eosinophil counts significantly decreased. Flow cytometry analysis of peripheral blood mononuclear cells in patients before and after treatment showed a significant increase in Th1/Th2 and Treg/Th17 ratios compared to baseline. ELISA detection of peripheral blood cytokines in patients before and after treatment showed a decreasing trend in the levels of Th2‐related cytokine IL‐4 and Th17‐related cytokine IL‐17A in the patient's serum compared to baseline (Table 6, Figure S1A,B).

**TABLE 6:** Laboratory results before and after treatment.

Variable	Baseline (*n* = 34)	End of treatment (*n* = 23)	*p*
Baseline blood eosinophil count, median (IQR), 10^9^/L	0.3 (0.2, 0.5)	0.04 (0.03, 0.06)	< 0.001***
Total IgE level, median (IQR), IU/mL	266.4 (31.8, 1113.0)	145.9 (40.6, 1484.0)	0.831
Th1/Th2 ratio, median (IQR)	0.8 (0.4, 1.2)	1.0 (0.8, 1.4)	0.039*
Treg/Th17 ratio, median (IQR)	0.6 (0.5, 1.1)	1.2 (0.6, 1.9)	0.026*
IL‐4, median (IQR)	4.3 (3.6, 6.3)	3.8 (2.9, 4.8)	0.098
IL‐17A, median (IQR)	14.4 (10.4, 19.5)	11.3 (7.7, 17.7)	0.062

Abbreviations: IQR, interquartile range; SCH, stratum corneum hydration; TEWL, trans‐epidermal water loss.

* *p* < 0.05.

*** *p* < 0.001.

#### 3.1.4 Safety Analysis

Safety assessments were conducted using a combination of physical examinations, laboratory tests, and patient‐reported AEs. All AEs were tracked using a dedicated record, and the principal investigator was responsible for their adjudication. Two AEs were reported during the trial, both of which were cold and deemed not to be associated with treatment. No laboratory abnormalities were observed in any patient.

### 3.2 Network Pharmacology Predicts the Possible Interaction of AESS for AD

**FIGURE 2:**
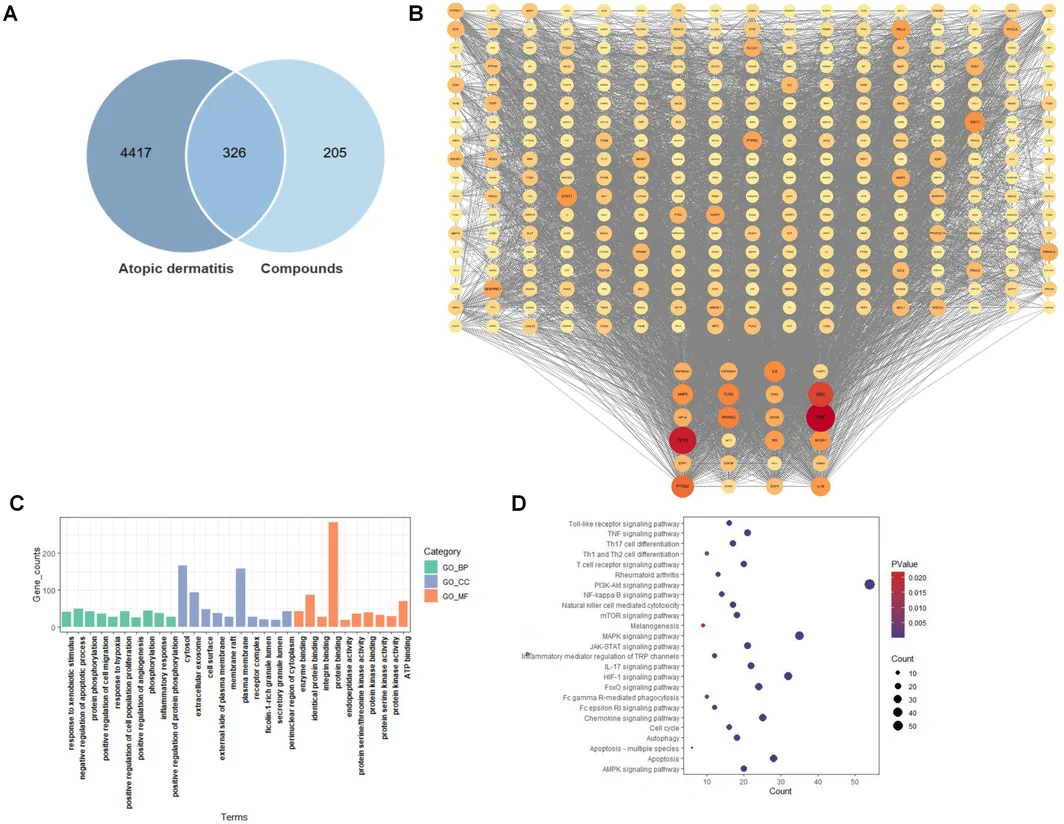
Network pharmacology prediction of gu‐ben‐hua‐shi (AESS) for atopic dermatitis (AD). (A) Venn diagram of common target genes between AESS compounds and AD. (B) The protein–protein interaction (PPI) network of the targets. (C) GO analysis of the potential functions of AESS. (D) Kyoto Encyclopedia of Genes and Genomes (KEGG) enrichment analysis of the potential pathways involved in AESS.

Integrated serum pharmacochemistry and network pharmacology were used to explore the mechanisms through which AESS alleviates dermatitis. Based on the top ten components identified in the AESS‐containing serum of rats (Table 7), 532 component targets and 4743 disease targets were obtained from the databases, with 326 overlapping targets (Figure 2A). Cytoscape v3.10.0 software was used to construct the PPI network. The results revealed 30 potential core targets of this formula for the treatment of AESS, including TNF, TP53, SRC, IL‐6, PPARG, TLR4, PTGS2, and mTOR (Figure 2B). GO and KEGG functional enrichment was analysed by using the R software with *p* < 0.05 for common targets. The 10 items in each category and 25 KEGG pathways were analysed using histograms and bubble plots for visualization (Figure 2C,D). Biological process (BP) enrichment mainly involved the regulation of cell migration, protein phosphorylation, and inflammatory response. Cellular component (CC) enrichment mainly included extracellular exosomes, receptor complexes, and ficolin‐1‐rich granule lumen. Molecular function (MF) enrichment analysis revealed enzyme binding, protein binding, and protein kinase activities (Figure 2C). The KEGG results showed involvement mainly in the PI3K‐Akt (hsa04151), mTOR (hsa04150), MAPK (hsa04010), Th17 cell differentiation (hsa04659), NF‐κB (hsa04064), and autophagy (hsa04140) signalling pathways (Figure 2D).

**TABLE 7:** Phytochemical components identified in gu‐ben‐hua‐shi (AESS) containing serum of rats.

No.	Components	2D structure	PubChem CID	Formula	CAS
1	Neochlorogenic acid	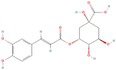	5,280,633	C16H18O9	996‐33‐2
2	Chlorogenic acid	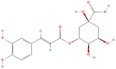	1,794,427	C16H18O9	327‐97‐9
3	Cryptochlorogenic acid	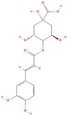	9,798,666	C16H18O9	905‐99‐7
4	Prim‐O‐glucosylcimifugin	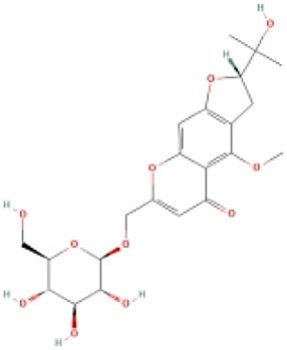	14,034,912	C22H28O11	80681‐45‐4
5	Rutin	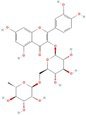	5,280,805	C27H30O16	153‐18‐4
6	Kaempferol‐3‐O‐rutinoside	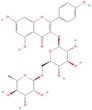	5,318,767	C27H30O15	17650‐84‐9
7	Narcissoside	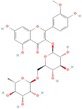	5,481,663	C28H32O16	604‐80‐8
8	Isochlorogenic acid A	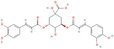	6,474,310	C25H24O12	2450‐53–5
9	5‐O‐Methylvisammioside	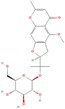	21,670,038	C22H28O10	84272‐85‐5
10	Isochlorogenic acid C	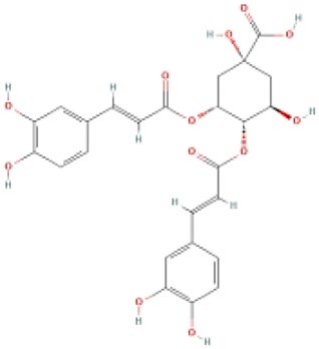	6,474,309	C25H24O12	57378‐72‐0/32451‐88‐0

### 3.3 AESS Alleviates Rash in AD Mice, and Its Efficacy Is Weakened After YAP Knockdown

**FIGURE 3:**
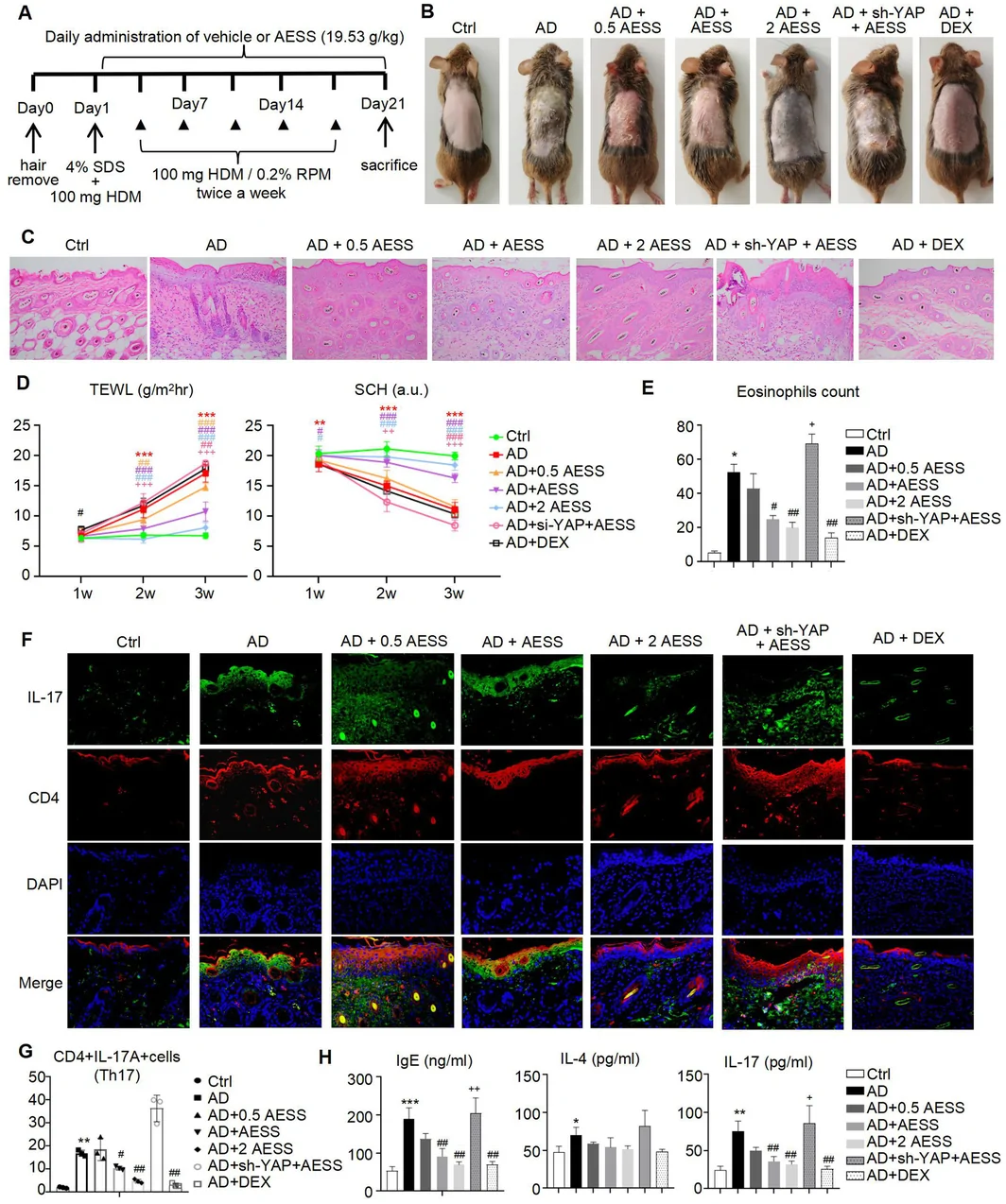
The effect of gu‐ben‐hua‐shi (AESS) on skin lesions and immune balance in atopic dermatitis (AD) mice models. (A) HDM‐induced AD mouse modelling scheme. (B) General image of mice. (C) Haematoxylin and eosin staining of the histopathological features (200×). (D) Trans Epidermal Water Loss (TEWL) and stratum corneum hydration (SCH) of each group. (E) Eosinophil counts after histopathological HE staining. (F) Immunofluorescence results for CD4 + IL‐17A + cells (Th17) (400×). IL‐17: green. CD4: red. (G) CD4 + IL‐17A+ cell (Th17) count determined by immunofluorescence staining. (H) IgE, IL‐4, and IL‐17 levels in mouse serum were determined by enzyme‐linked immunosorbent assay. Data are shown as the mean ± standard deviation (SD) of at least three independent experiments (*n* = 6). *p < 0.05, **p < 0.01, ***p < 0.001 AD versus Ctrl; #*p* < 0.05, ##*p* < 0.01 versus AD; +*p* < 0.05, ++*p* < 0.01 AD + sh‐YAP + AESS versus AD + AESS. AD + 0.5 AESS, mice intragastrically administered of 1/2 of the standard dose of AESS formula; AD + 2 AESS, mice intragastrically administered of 2 times of the standard dose of AESS formula; AD + AESS, mice intragastrically administered the standard dose of AESS formula; AD + DEX, mice intragastrically administered 3 mg/kg dexamethasone; AD + sh‐YAP + AESS, mice of the AD + AESS group injected with shRNA‐YAP lentivirus; AD, atopic dermatitis model mice; Ctrl, control mice.

The AD model induced by dust mites (Figure 3A) was very successful, and the rash tissue of the mice was clinically and pathologically similar to that of humans. There were no significant differences in body weight between all groups of mice, and there were no significant differences in the rash score, ear thickness, TEWL, or SCH between mice of different sexes (Table S3–S5). Compared to the AD model group, oral administration of the AESS formula resulted in milder skin lesions, decreased dermatitis scores, reduced ear swelling, decreased TEWL, and increased SCH. However, injecting YAP shRNA lentivirus into the AESS group reversed these results. The positive drug dexamethasone damaged the skin barrier, accompanied by an increase in TEWL and a decrease in SCH, which was not significantly different from that in the AD group (Figure 3B,D, Table 8).

**TABLE 8:** Dermatitis score, skin lesion thickness, and ear thickness in different groups of mice.

Group	Dermatitis score	Skin lesion thickness (μm)	Ear thickness (mm)
Ctrl	0	23.523 ± 1.743	0.152 ± 0.010
AD	3.833 ± 1.169**	129.631 ± 7.027***	0.208 ± 0.015***
AD + 0.5 AESS	4.000 ± 0.894	112.800 ± 12.587	0.202 ± 0.008
AD + AESS	1.833 ± 0.753	68.230 ± 8.987^a^	0.180 ± 0.006^a^
AD + 2 AESS	1.333 ± 0.516*	52.780 ± 6.198^a^	0.168 ± 0.008^a^
AD + sh‐YAP + AESS	4.167 ± 0.983^c^	184.901 ± 17.020^a^, ^b^	0.237 ± 0.012^a^, ^b^
AD + DEX	1.167 ± 0.408*	33.137 ± 7.410^a^	0.162 ± 0.012^a^

Abbreviations: AD + 0.5 AESS, mice intragastrically administered of 1/2 of the standard dose of AESS formula; AD + 2 AESS, mice intragastrically administered of 2 times of the standard dose of AESS formula; AD + AESS, mice intragastrically administered the standard dose of AESS formula; AD + DEX, mice intragastrically administered 3 mg/kg dexamethasone; AD + sh‐YAP + AESS, mice of the AD + AESS group injected with shRNA‐YAP lentivirus; AD, atopic dermatitis model mice; Ctrl, control mice.

* *p* < 0.05 versus AD.

** *p* < 0.01 AD versus Ctrl.

*** *p* < 0.001 AD versus Ctrl.

^a^ *p* < 0.001 versus AD.

^b^ *p* < 0.001 versus AD + sh‐YAP + AESS versus AD + AESS.

^c^ *p* < 0.05 versus AD + sh‐YAP + AESS versus AD + AESS.

### 3.4 AESS Reduced Inflammatory Cell Infiltration and the Expression of Inflammatory Cytokines in AD Mice, and YAP Knockdown Weakened Its Efficacy

The number of eosinophils was counted after HE staining at high magnification, and the average of at least three counts under high magnification was calculated for each mouse. Additionally, we calculated the number of infiltrating CD4+IL‐17A+ double‐positive cells (Th17 cells) using immunofluorescence staining. Compared to the AD model group, eosinophil and Th17 cell infiltration decreased in the groups treated with one and two times the standard dose of AESS. Injection of YAP shRNA lentivirus into the AESS group reversed these eosinophil infiltration results. Dexamethasone significantly reduced the number of eosinophils and Th17 cells (Figure 3E,G).

ELISA was used to detect the expression of IgE, IL‐4, and IL‐17A in the serum of mice in each group. Administration of one and two times the standard dose of AESS lowered the IgE and IL‐17A expression levels; however, the injection of YAP shRNA lentivirus into the AESS group reversed these results. IL‐4 levels showed the same trend; however, the difference was not statistically significant (Figure 3H).

### 3.5 AESS Influence the Hippo‐YAP Signalling Pathway Activity in AD Mice

**FIGURE 4:**
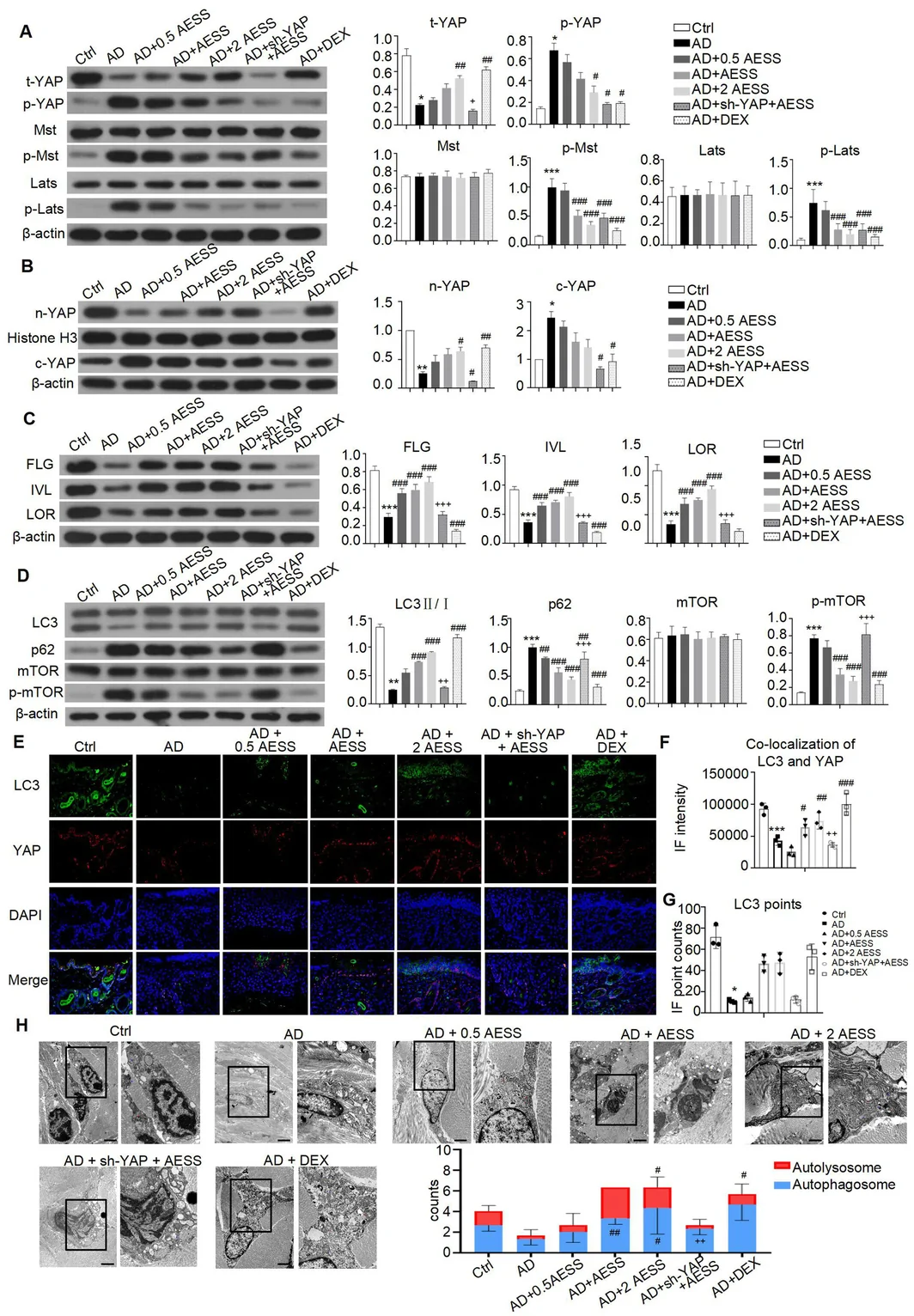
The effect of gu‐ben‐hua‐shi (AESS) on skin barrier proteins and autophagy in atopic dermatitis (AD) mice models. (A) Expression of core kinase cascade proteins of the Hippo pathway. (B) Expression of nuclear YAP (n‐YAP) and cytoplasmic YAP (c‐YAP) protein in nuclear and cytoplasmic fractions. (C) Expression of the skin barrier‐related proteins FLG, IVL, and LOR. (D) Expression of autophagy‐related proteins LC3, p62, mTOR, and p‐mTOR. (E) Immunofluorescence results for LC3 and YAP (400×). LC3: Green. YAP: Red. (F) LC3‐YAP co‐localization by immunofluorescence (400×). (G) LC3 positive cells were detected by immunofluorescence (600×). (H) Electron microscopy results of autolysosome and autophagosome counts (left: 10,000×; right: 20,000×, red arrows indicate autolysosome, blue arrows indicate autophagosomes; bar length = 2 μm). Data are shown as the mean ± standard deviation (SD) of at least three independent experiments (*n* = 6). *p < 0.05, **p < 0.01, ***p < 0.001 AD versus Ctrl; #*p* < 0.05, ##*p* < 0.01, ###*p* < 0.001 versus AD; +*p* < 0.05, ++*p* < 0.01, +++*p* < 0.01 AD + sh‐YAP + AESS versus AD + AESS. AD + AESS, mice intragastrically administered the standard dose of AESS formula; AD + DEX, mice intragastrically administered 3 mg/kg dexamethasone; AD + 2 AESS, mice intragastrically administered of 2 times of the standard dose of AESS formula; AD + sh‐YAP + AESS, mice of the AD + AESS group injected with shRNA‐YAP lentivirus; AD, atopic dermatitis model mice; AD + 0.5 AESS, mice intragastrically administered of 1/2 of the standard dose of AESS formula; Ctrl, control mice.

In an AD mouse model, YAP expression decreased, whereas p‐Mst, p‐Lats, and p‐YAP expression increased. After gastric lavage with AESS, YAP expression in mice was restored in a dose‐dependent manner. Although the expression levels of Mst and Lats remained unchanged, those of p‐Mst, p‐Lats, and p‐YAP decreased. However, changes in p‐Mst and p‐Lats levels in the YAP shRNA lentivirus‐injected group were not significant (Figure 4A). The level of nuclear YAP (n‐YAP) improved in a dose‐dependent manner, and that of cytoplasmic YAP (c‐YAP) decreased after the oral administration of AESS, while after the injection of YAP shRNA this trend decreased but was not significantly different from that in the AD group (Figure 4B). Immunofluorescence results showed the same trend, which showed significant YAP nuclear accumulation (Figure S2A). As both Mst and Lats are located upstream of YAP in the Hippo signalling pathway, MST1/2 undergoes phosphorylation, thereby mediating the phosphorylation of LAST1/2. p‐LAST1/2 exhibits kinase activity and further mediates YAP phosphorylation, whereas p‐YAP is inactive and remains in the cytoplasm [31]. These results indicate that AESS exerts its effects by inhibiting the activity of the Hippo signalling pathway and suppressing the phosphorylation of MST1/2, LAST1/2, and YAP in the core part of the kinase cascade, as well as induced YAP nuclear translocation.

### 3.6 AESS Increases the Expression of Barrier Proteins in AD Mice, but Its Efficacy Is Reversed After YAP Knockdown

Western blotting was used to detect the expression of FLG, IVL, and LOR in tissues. The expression of FLG, IVL, and LOR increased in a dose‐dependent manner after the oral administration of AESS. Injection of YAP shRNA lentivirus into the AESS group reduced the expression of FLG, IVL, and LOR in mouse skin lesions. Dexamethasone significantly reduced the expression levels of FLG, IVL, and LOR, indicating its ability to break down the skin barrier (Figure 4C).

### 3.7 AESS Enhances Autophagy in AD Mice, but Its Efficacy Is Reversed After YAP Knockdown

Western blotting was used to detect the expression of LC3‐II/I and p62 in the mouse skin lesions. The expression of LC3‐II/I increased, and that of p62 decreased in a dose‐dependent manner in mice after oral administration of the AESS formula and dexamethasone. Injection of the YAP shRNA lentivirus into the AESS group reversed these results. In the autophagy inhibitory pathway, the mTOR signalling pathway, p‐mTOR activity was partially reduced after gavage with the AESS formula and positive drug dexamethasone and increased after injection of the YAP shRNA lentivirus (Figure 4D).

Immunofluorescence staining results revealed an increase in the co‐localization of YAP and LC3 after one and two times the standard dosage of AESS or dexamethasone treatment in the AD mouse model, which was reduced after injection of the YAP shRNA lentivirus. The number of LC3 fluorescent points showed the same trend; however, the difference was not statistically significant (Figures 4E–G and S2B).

Transmission electron microscopy revealed a decrease in the autolysosome and autophagosome count in AD mice, followed by an increase after administration of one and two times the standard dose of AESS or dexamethasone. While this trend decreased again after the injection of YAP shRNA (Figure 4H).

### 3.8 AESS Increases Proliferation and Decreases Apoptosis in AD‐Like Inflammatory Cell Models, and Knocking Out YAP Reverses the Effect

**FIGURE 5:**
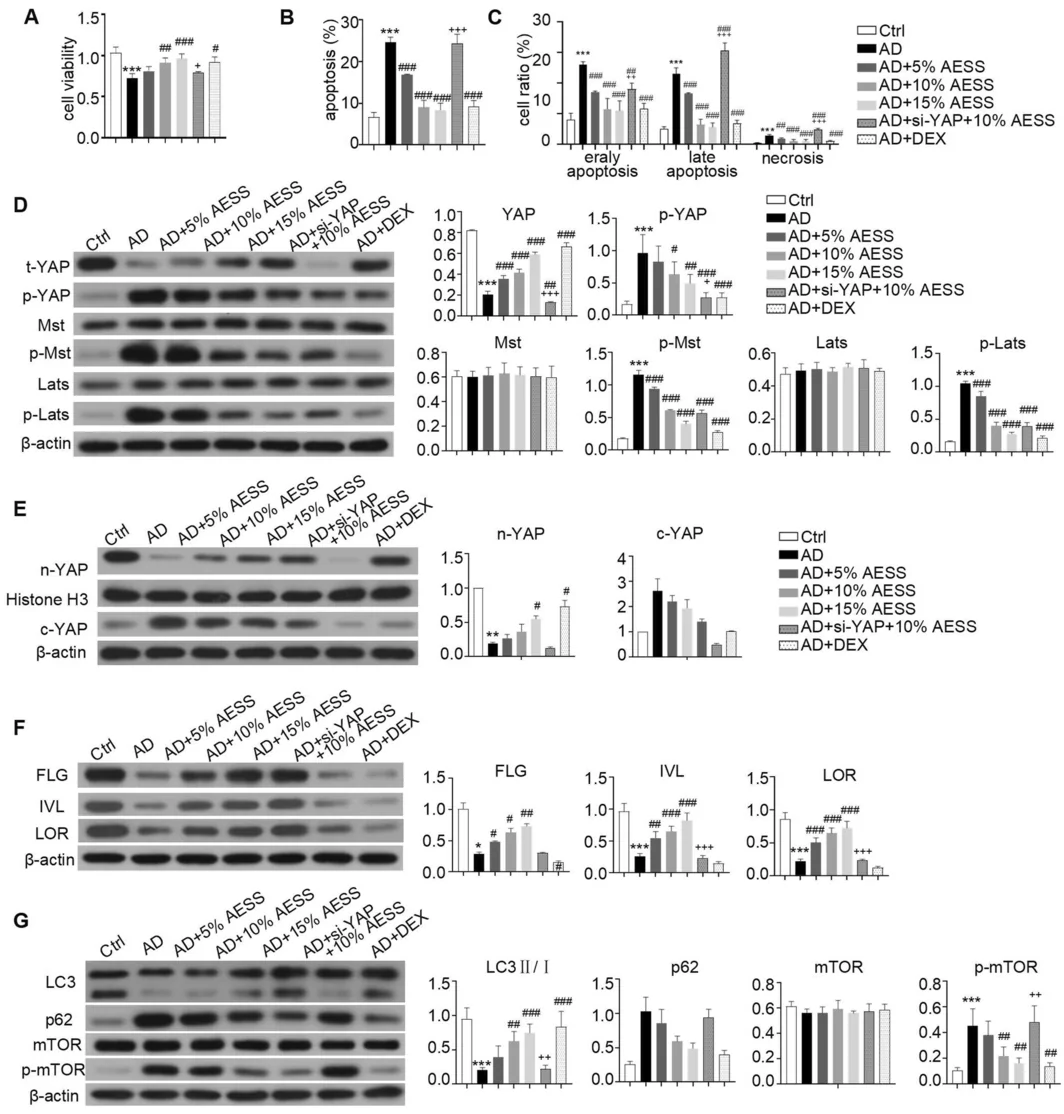
The effect of gu‐ben‐hua‐shi (AESS) on atopic dermatitis (AD) like inflammatory cell model. (A) Cell viability was determined using the MTT assay. (B) Total cell apoptosis rate was determined by flow cytometry. (C) Flow cytometric analysis of early and late apoptosis and necrosis rates. (D) Expression of core kinase cascade proteins of the Hippo pathway. (E) Expression of YAP protein in nuclear and cytoplasmic fractions. (F) Expression of the skin barrier‐related proteins FLG, IVL, and LOR. (G) Expression of autophagy‐related proteins LC3, p62, mTOR, and p‐mTOR. *p < 0.05, **p < 0.01, ***p < 0.001 ad versus Ctrl; #*p* < 0.05, ##*p* < 0.01, ###*p* < 0.001 versus AD; +*p* < 0.05, ++*p* < 0.01, +++*p* < 0.001 AD + si‐YAP + 10% AESS versus AD + 10% AESS. AD, atopic dermatitis like inflammatory cells; AD + 10% AESS, AD‐like cells cultured with 10% AESS contained serum; AD + 15% AESS, AD‐like cells cultured with 15% AESS contained serum; AD + 5% AESS, AD‐like cells cultured with 5% AESS contained serum; AD + DEX, AD‐like cells cultured with 20 μg/mL dexamethasone; AD + si‐YAP + 10% AESS, cells of the AD+10% AESS group transfected with YAP si‐RNA; Ctrl, control cells.

MTT assay revealed a decrease in the proliferative ability of AD‐like inflammatory cells. The proliferative ability of the cells increased after treatment with one and two times the standard dose of AESS serum or dexamethasone. YAP‐knockdown and culture with AESS serum reduced the proliferative ability of the cells (Figure 5A).

Flow cytometry revealed an increase in the apoptosis of AD‐like inflammatory cells. After treatment with serum AESS or dexamethasone, the number of apoptotic cells decreased. After YAP knockdown, the total apoptotic rate of cells increased, including an increase in both early and late apoptosis (Figures 5B,C and S3A).

### 3.9 AESS Can Alter the Activity of Hippo‐YAP Signalling Pathway in AD‐Like Inflammatory Cells

Western blotting results showed decreased YAP expression and increased p‐Mst, p‐Lats, and p‐YAP expression in the AD cell model. YAP expression in the cells was restored after treatment with serum containing AESS or dexamethasone. Although the expression levels of Mst and Lats remained unchanged, those of p‐Mst, p‐Lats, and p‐YAP decreased. However, changes in p‐Mst, p‐Lats, and p‐YAP levels in the YAP knockdown group were not significant (Figure 5D). The localisation of n‐YAP improved after treatment with serum AESS or dexamethasone, while the level of c‐YAP and the phosphorylation of Hippo pathway components reduced. However, changes in n‐YAP and c‐YAP levels in the YAP shRNA lentivirus‐injected group were not significant (Figure 5E). Immunofluorescence also revealed that the increased n‐YAP fluorescence after culturing with AESS‐containing serum (Figure S3B). These results are consistent with those in mice, indicating that the target of AESS may be located upstream of the Hippo signalling pathway.

### 3.10 AESS Increases the Expression of Barrier Proteins in AD‐Like Inflammatory Cells, but the Efficacy Is Weakened After YAP Knockdown

Western blotting revealed increased expression of FLG, IVL, and LOR following AESS treatment. YAP knockdown and AESS treatment significantly reduced the expression of FLG, IVL, and LOR in these cells. Dexamethasone treatment significantly reduced the FLG expression (Figure 5F). These results are consistent with those obtained in mice, indicating that AESS restores the expression of skin barrier proteins via YAP.

### 3.11 AESS Increases the Autophagy Level of AD‐Like Inflammatory Cells, and the Efficacy Is Reversed After YAP Knockdown

Western blotting revealed increased LCII/I expression and decreased p‐mTOR expression after treatment with AESS or dexamethasone. YAP knockdown reduced the expression of LC3‐II/I and increased p‐mTOR expression. The expression of p62 showed a similar trend, but the difference was not statistically significant (Figure 5G).

**FIGURE 6:**
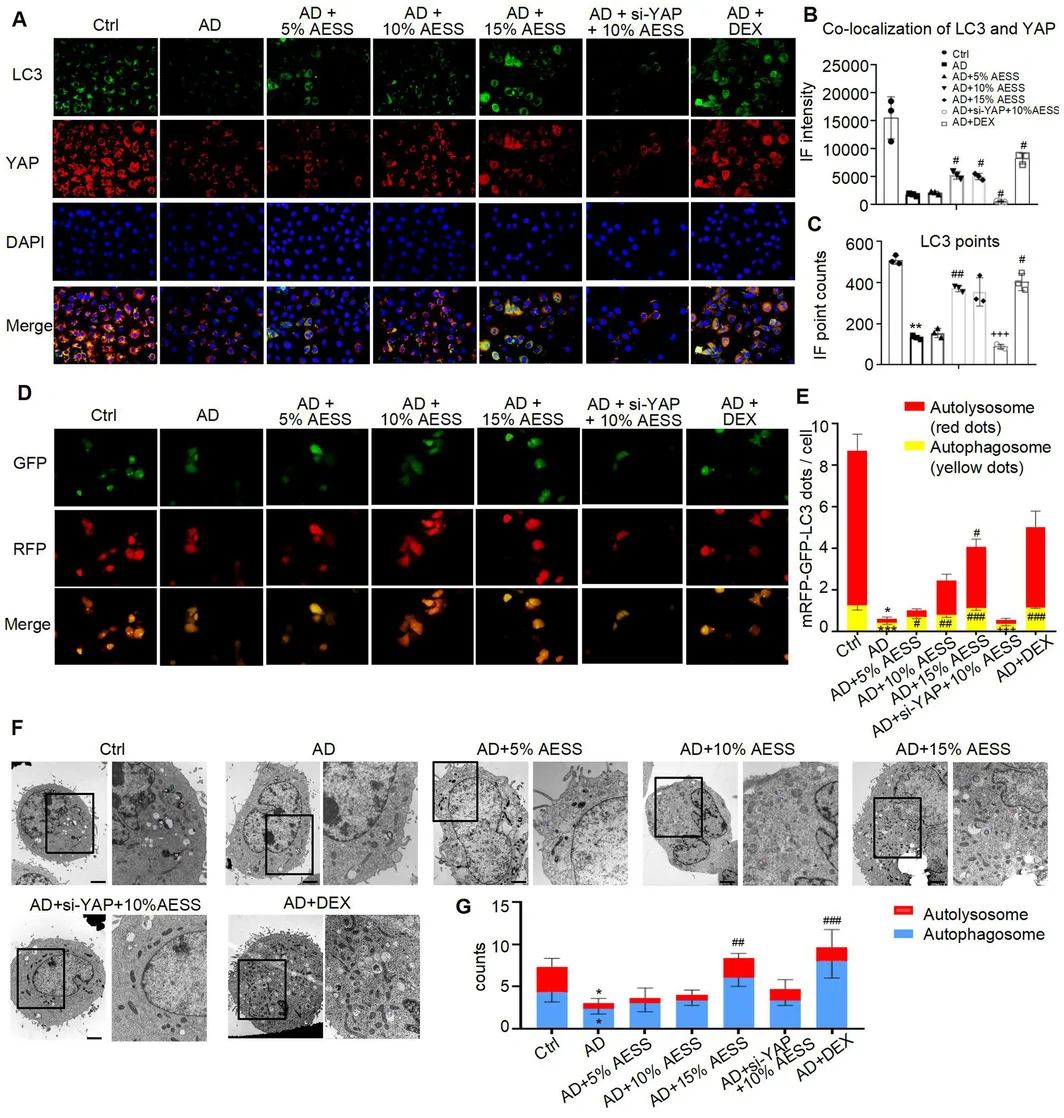
The effect of gu‐ben‐hua‐shi (AESS) on atopic dermatitis (AD) like inflammatory cell model. (A) Immunofluorescence results for LC3 and YAP (400×). LC3: green. YAP: red. (B) LC3‐YAP co‐localization by immunofluorescence (400×). (C) LC3 positive cells were identified by immunofluorescence (600×). (D, E) Tandem mRFP‐GFP‐LC3 fluorescent results for autophagy flux (400×). (F, G) Electron microscopy results of autolysosome and autophagosome counts (left: 10,000×; right: 20,000×, red arrows indicate autolysosome, blue arrows indicate autophagosomes; bar length = 2 μm). Data are shown as the mean ± standard deviation (SD) of at least three independent experiments. *p < 0.05, **p < 0.01, ***p < 0.001 AD versus Ctrl; #*p* < 0.05, ##*p* < 0.01, ###*p* < 0.001 versus AD; +*p* < 0.05, +++*p* < 0.001 AD + si‐YAP + 10% AESS versus AD + 10% AESS. AD + 10% AESS, AD‐like cells cultured with 10% AESS contained serum; AD + 15% AESS, AD‐like cells cultured with 15% AESS contained serum; AD + 5% AESS, AD‐like cells cultured with 5% AESS contained serum; AD + DEX, AD‐like cells cultured with 20 μg/mL dexamethasone; AD + si‐YAP + 10% AESS, cells of the AD + 10% AESS group transfected with YAP si‐RNA; AD, atopic dermatitis like inflammatory cells; Ctrl, control cells.

Immunofluorescence staining showed increased LC3 fluorescence and co‐localization of LC3 and YAP in the AD cell model after AESS or dexamethasone treatment. After YAP knockdown and treatment with AESS, the number of LC3 fluorescence points and the co‐localization of LC3 and YAP decreased (Figures 6A–C and S3C).

Tandem mRFP‐GFP‐LC3 fluorescence analysis revealed a significant decrease in autolysosome and autophagosome dots in the AD cell model, while increased after AESS or dexamethasone treatment. YAP knockdown decreased the number of autophagosomes and autolysosomes, indicating that AESS promotes autophagic flux (Figure 6D,E).

Transmission electron microscopy showed a significant decrease in autolysosomes and autophagosome counts in the AD cell model, followed by an upward trend after culturing with AESS‐containing serum. After YAP knockdown, this trend decreased but was not statistically significant (Figure 6F,G). These results were consistent with those obtained in mice, indicating that AESS plays a role in restoring AD autophagy through YAP.

## 4 Discussion

Atopic dermatitis (AD) is a chronic skin disease characterized by skin barrier dysfunction and immune balance disorders. In this study, we evaluated the effects of AESS on the skin barrier and its underlying mechanisms in alleviating AD. The single‐arm clinical trial analysed the therapeutic effects of AESS in patients with AD. The results showed that most of the included patients had moderate‐to‐severe AD. After intervention with AESS, the severity of the disease and subjective symptoms significantly improved. SCORAD, IGA, and EASI scores decreased over time. In animal models, the dermatitis score, ear thickness, and skin lesion thickness of mice treated with AESS improved, indicating that AESS can improve skin lesions and alleviate symptoms in patients. A network pharmacology study was conducted to further explore the potential targets and mechanistic pathways of AESS, and the results showed that the therapeutic effect of AESS on AD involved multiple targets, which are closely associated with Hippo‐YAP and autophagy [32, 33, 34].

Laboratory test results indicate a decreasing trend in immunoglobulin IgE and eosinophil levels in patients after treatment, suggesting the clinical effectiveness of AESS in improving the progression of AD. The levels of the serum Th17‐related cytokine IL‐17A and Th2‐related cytokines IL‐4 and IL‐13 showed a decreasing trend, whereas the ratios of Th1/Th2 and Treg/Th17 significantly increased. In animal experiments, the infiltration of Th17 cells decreased in mice treated with AESS, as did the expression of serum IgE, IL‐4, and IL‐17A. AESS has anti‐inflammatory effects, which may be achieved by regulating Th2 and Th17 cells. Immune dysfunction plays an important role in the initiation and progression of AD. A Th1/Th2 cell immune imbalance is a classic pathogenesis of AD. Th2 cells promote IgE overexpression by secreting IL‐4, which triggers a series of immune responses in the body [35]. Th17 cells and their related cytokine IL‐17 play an important role in the pathogenesis of AD. Previous studies showed that the IL‐17 pathway is upregulated in Asian populations [36] further explored suggesting that Th1, Th2, and Th17 cells in healthy skin tissue, acute lesional tissue, and chronic lesional tissue were all involved in the inflammatory response and showed a gradually increasing trend. FLG can be broken down into natural moisturizing molecules that play an important role in maintaining skin water content and the barrier function of the stratum corneum. During the occurrence and development of AD, Th2 cell overexpression may result in the downregulation of FLG, particularly IL‐4 and IL‐13 [37]. Excessive IL‐17 can disrupt the expression of tight junction proteins in the skin barrier and inhibit the processing of FLG from precursor proteins to amino acids [38], indicating that immune balance disorders, especially the Th2 and Th17 immune responses, are involved in the disruption of the skin barrier in AD. These results suggest that the regulatory effects of AESS on immune balance disorders can ameliorate skin barrier damage.

A comparison of skin physiological parameters at baseline and week 8 revealed that SCH in lesioned and nonlesioned areas increased significantly over time, indicating enhanced skin hydration and improved dryness after treatment. The TEWL rate in the lesion area significantly improved at week 8. The improvement in TEWL in animal experiments is consistent with this, and both animal and cell experiments have demonstrated that AESS can increase the expression of the epidermal barrier proteins FLG, IVL, and LOR, confirming that AESS can effectively improve skin barrier function. Reduced autophagy is involved in the pathogenesis of skin barrier dysfunction in patients with AD, playing a regulatory role in inflammatory responses, keratinocyte differentiation, and host defence against invading pathogens, such as *Staphylococcus aureus* . Inhibiting the expression of autophagic proteins can suppress FLG expression, indicating that epidermal barrier function is reduced when autophagy is inhibited. mTOR is an inhibitor of autophagy [39]. The results of this study also demonstrate that AESS can enhance autophagy in local keratinocytes in AD lesions by inhibiting the mTOR pathway, thereby playing a role in restoring the skin barrier.

**FIGURE 7:**
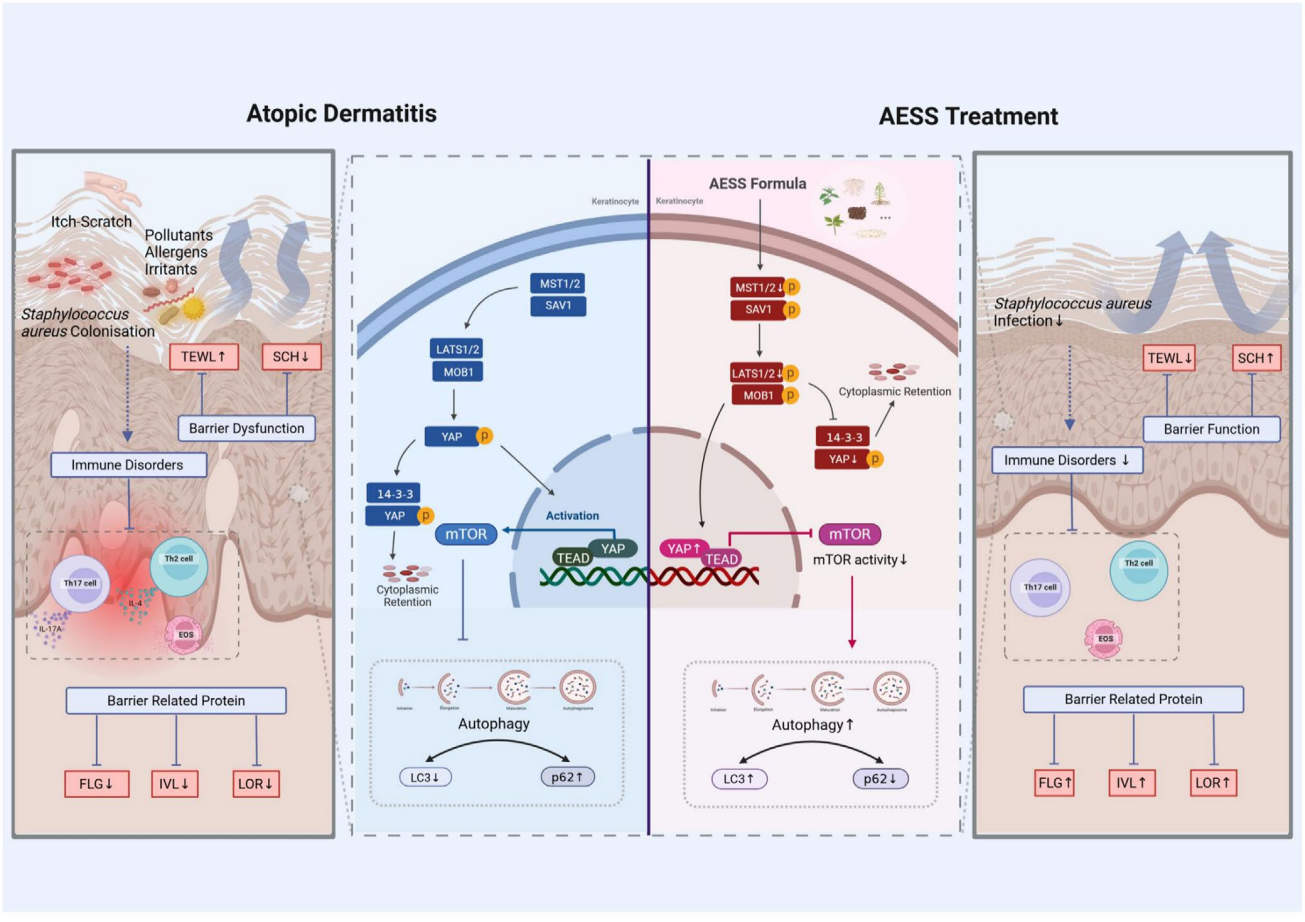
The mechanism of gu‐ben‐hua‐shi (AESS) in the treatment of atopic dermatitis (AD) through the Hippo–YAP pathway.

YAP is a key member downstream of the Hippo signalling pathway, which plays a role in regulating the proliferation and differentiation of epidermal keratinocytes, wound healing, maintaining the three‐dimensional structure of the skin, and maintaining cellular autophagy function [14, 15, 40]. Our previous study showed that AESS increases YAP expression in AD keratinocytes [12]. This study further found that AESS inhibited the phosphorylation of core components of the Hippo kinase cascade, such as MST1/2, LAST1/2, and YAP, in animal and cell models. After the inhibition of YAP expression and treatment with AESS, the expression of barrier‐related proteins and autophagy levels decreased significantly, indicating that the recovery effect of AESS on the AD skin barrier was achieved through the Hippo‐YAP signalling pathway (Figure 7).

It has been clinically demonstrated that AESS can be administered simultaneously with conventional AD treatments, including topical corticosteroids, topical calcineurin inhibitors and oral antihistamines. Previous studies have confirmed that herbal medicines, along with conventional treatments, can enhance treatment efficacy [41, 42]. Prioritizing scientific rigour, topical drugs affect the skin barrier and treatment efficacy. Therefore, this study did not include drugs that were used simultaneously. However, AESS alone has been shown to exert therapeutic effects. According to the principles of TCM, the interaction between an overactive heart fire or embedded dampness and spleen deficiency is the main pathogenesis of AD and should be targeted in treatment to alleviate symptoms [6]. The spleen governs water metabolism, and its dysfunction induces the retention of dampness and impairment of the skin barrier, which correlate with disordered keratinocyte autophagy and YAP activation. Regulating the Hippo‐YAP‐autophagy mechanism invigorates the spleen, eliminates dampness and achieves therapeutic effects. This study has some limitations. This was an exploratory pilot short‐term intervention study owing to the lack of prior reference data; therefore, it has limitations in terms of study design and data collection, and follow‐up and observation are still required. A placebo‐controlled, RCT with more comprehensive evaluation criteria is currently in progress.

## 5 Conclusions

Our study proved that AESS can improve the skin barrier and simultaneously regulate immune balance disorders, resolving the two main mechanisms of AD with minimal side effects by involving the Hippo‐YAP‐mediated autophagy pathway, thus providing a good opportunity for the treatment of AD with Chinese herbal medicine. However, considering the complexity of intercellular signal transduction and the cross‐interactions between different signalling pathways, more research is needed to confirm the conclusions of this study and verify whether AESS also affects other pathway mechanisms.

## Author Contributions

**Xin Ma:** writing – original draft, methodology, data curation, investigation. **Yiting Huang:** methodology, investigation. **Xiao Lv:** methodology, investigation. **Xiumei Mo:** investigation, validation, formal analysis. **Junfeng Liu:** investigation, validation, formal analysis. **Fenggen Yan:** methodology, investigation. **Siqi Ye:** methodology, investigation. **Yu Zhang:** investigation, data curation. **Dacan Chen:** conceptualization, supervision, project administration, resources. **Jinjing Jia:** conceptualization, investigation, methodology, supervision, funding acquisition, writing – review and editing, validation.

## Funding

This work was supported by the National Natural Science Foundation of China (82274521), the Guangzhou Basic and Applied Basic Research Foundation (2024A03J0547; 2025A03J4063), Research Project of Guangdong Provincial Bureau of Traditional Chinese Medicine (20254057), the Research Fund for Qingmiao Talents of Guangdong Provincial Hospital of Chinese Medicine (SZ2024QN04), the Guangdong Provincial Key Laboratory of Chinese Medicine for prevention and treatment of Refractory Chronic Disease (2023KT15528), and the State Key Laboratory of Traditional Chinese Medicine Syndrome Member Project (QZ2025ZZ24).

## Conflicts of Interest

The authors declare no conflicts of interest.

## Supporting information


**Figure S1:** The effect of gu‐ben‐hua‐shi (AESS) on inflammatory disorder in atopic dermatitis (AD) patients. (A) Flow cytometry results from the clinical patients before/after AESS treatment. (B) Scatter plot of IL‐4, IL‐17A, Th1/Th2 and Th17/Treg of the clinical patients before/after AESS treatment. *p < 0.05 versus. Baseline.
**Figure S2:** The effect of gu‐ben‐hua‐shi (AESS) on autophagy in atopic dermatitis (AD) mice models. (A) Immunofluorescence analysis of YAP subcellular localization. (B) LC3 positive cells in mice skin lesions from immunofluorescence (left: 400×; middle: 600×; right: 1000×, red arrows indicate LC3 fluorescent points).
**Figure S3:** The effect of gu‐ben‐hua‐shi (AESS) on atopic dermatitis (AD) like inflammatory cell model. (A) Flow cytometry analysis of cell apoptosis rate results. (B) Immunofluorescence analysis of YAP subcellular localization. (C) LC3 positive cells in cells from immunofluorescence (left: 400×; middle: 600×; right: 1000×, red arrows indicate LC3 fluorescent points).
**Table S1:** Chemical compounds of AESS.
**Table S2:** The sequences of siRNAs/shRNAs used in this study.
**Table S3:** The dermatitis score and ear thickness of the mice.
**Table S4:** Trans‐epidermal water loss (TEWL) of the mice (g/m2h).
**Table S5:** Stratum corneum hydration (SCH) of the mice (a.u.).

## Data Availability

The data that support the findings of this study are available from the corresponding author upon reasonable request.
