# Supplementary material for: Mechanism of Gu‐Ben‐Hua‐Shi (AESS) Formula Regulating Autophagy Through Hippo‐YAP Pathway in Restoring the Skin Barrier in Atopic Dermatitis
Source: J Cell Mol Med. 2026 Sep 12;30(17):e71361. doi: 10.1111/jcmm.71361 (PMC13570263; doi:10.1111/jcmm.71361)
Supplement: Supplementary file 1 [file JCMM-30-e71361-s001.docx]

**Supplementary Tables**

**Table S1**

Chemical compounds of AESS.

| No | Compound | Formula | Classification | Origin |
| --- | --- | --- | --- | --- |
| 1 | Citric Acid | C_6_H_8_O_7_ | Organic acids | Atractylodis Macrocephalae Rhizom |
| 2 | Adenosine | C_10_H_13_N_5_O_4_ | Nucleoside | Coicis Semen |
| 3 | L-Phenylalanine | C_9_H_11_NO_2_ | Amino acids | Atractylodis Rhizoma |
| 4 | Protocatechuic acid | C_7_H_6_O_4_ | Organic acids | Sophorae Flos |
| 5 | Leonuride | C_15_H_24_O_9_ | Terpenoids | Rehmanniae Radix |
| 6 | 5-hydroxymethyl furfural | C_6_H_6_O_3_ | Furaldehyde | Artificial derivative |
| 7 | Neochlorogenic acid | C_16_H_18_O_9_ | Organic acids | Lonicerae Japonicae Flos |
| 8 | D-tryptophan | C_11_H_12_O_2_N_2_ | Amino acids | Atractylodis Rhizoma |
| 9 | Protamine sulfate | C_7_H_6_O_3_ | Organic acids | Lonicerae Japonicae Flos |
| 10 | Salidroside | C_14_H_20_O_7_ | Glycosides | Lonicerae Japonicae Flos |
| 11 | 8-Epi-Loganic acid | C_16_H_24_O_10_ | Terpenoids | Rehmanniae Radix |
| 12 | Unknown | C_18_H_26_O_13_ | Unknown | Lonicerae Japonicae Flos |
| 13 | Loganic acid | C_16_H_24_O_10_ | Terpenoids | Lonicerae Japonicae Flos |
| 14 | Atractyloside A | C_21_H_36_O_10_ | Glycosides | Atractylodis Rhizoma |
| 15 | (1R,4S,6R)-1,3,3-trimethyl-2-oxabicyclo[2.2.2]oct-6-yl-6-O-β-D-glucopyranosyl-β-D-glucopyranoside | C_22_ H_38_ O_12_ | Glycosides | Atractylodis Macrocephalae Rhizom, Atractylodis Rhizoma |
| 16 | Chlorogenic acid | C_16_H_18_O_9_ | Organic acids | Lonicerae Japonicae Flos |
| 17 | Morroniside | C_17_H_26_O_11_ | Terpenoids | Rehmanniae Radix |
| 18 | Cryptochlorogenic acid | C_16_ H_18_ O_9_ | Organic acids | Lonicerae Japonicae Flos |
| 19 | Unknown | C_16_ H_24_ O_10_ | Unknown | Lonicerae Japonicae Flos |
| 20 | Xanthoxylin | C_10_ H_12_ O_4_ | Phenols | Lonicerae Japonicae Flos |
| 21 | Unknown | C_16_ H_22_ O_11_ | Unknown | Lonicerae Japonicae Flos |
| 22 | Secologanic acid | C_16_H_22_O_10_ | Terpenoids | Lonicerae Japonicae Flos |
| 23 | Unknown | C_21_H_32_O_14_ | Unknown | Lonicerae Japonicae Flos |
| 24 | Unknown | C_28_ H_43_O_20_ | Unknown | Lonicerae Japonicae Flos |
| 25 | Unknown | C_16_ H_17_O_9_ | Organic acids | Lonicerae Japonicae Flos |
| 26 | 5-*p*-coumaroylquinic acid | C_16_ H_18_O_8_ | Organic acids | Lonicerae Japonicae Flos |
| 27 | Unknown | C_19_ H_28_ O_12_ | Unknown | Lonicerae Japonicae Flos |
| 28 | Vegeloside | C_17_H_24_O_10_ | Terpenoids | Lonicerae Japonicae Flos |
| 29 | Loganin | C_17_H_26_O_10_ | Terpenoids | Lonicerae Japonicae Flos |
| 30 | Sweroside | C_16_H_22_O_9_ | Terpenoids | Lonicerae Japonicae Flos |
| 31 | RehMapicroside | C_16_H_26_O_8_ | Terpenoids | Rehmanniae Radix |
| 32 | 3-O-feruloylquinic acid | C_17_H_20_O_9_ | Organic acids | Lonicerae Japonicae Flos |
| 33 | Echinacoside | C_35_ H_46_ O_20_ | Phenylethanoid glycosides | Rehmanniae Radix |
| 34 | Unknown | C_17_H_26_O_10_ | Unknown | Lonicerae Japonicae Flos |
| 35 | Epivogeloside | C_17_H_24_O_10_ | Terpenoids | Lonicerae Japonicae Flos |
| 36 | Secoxyloganin | C_17_H_24_O_11_ | Terpenoids | Lonicerae Japonicae Flos |
| 37 | Cimicifugoside | C_22_ H_28_O_11_ | Chromones | Saposhnikoviae Radix |
| 38 | Jionoside A1 | C_36_ H_48_O_20_ | Phenylethanoid glycosides | Rehmanniae Radix |
| 39 | Unknown | C_26_H_35_O_11_ N | Unknown | Coicis Semen |
| 40 | Rutin | C_27_H_30_O_16_ | Flavonoids | Sophorae Flos |
| 41 | Isoquercitrin | C_21_H_20_O_12_ | Flavonoids | Sophorae Flos |
| 42 | Luteoloside | C_21_H_20_O_11_ | Flavonoids | Lonicerae Japonicae Flos |
| 43 | Unknown | C_25_H_31_O_4_N_3_ | Unknown | Coicis Semen |
| 44 | Lonicerin | C_27_H_30_O_15_ | Flavonoids | Lonicerae Japonicae Flos |
| 45 | Unknown | C_20_H_36_O_10_ | Unknown | Rehmanniae Radix |
| 46 | Verbascoside | C_29_H_36_O_15_ | Phenylethanoid glycosides | Rehmanniae Radix |
| 47 | Cimifugin | C_16_ H_18_ O_6_ | Chromones | Saposhnikoviae Radix |
| 48 | Kaempferol-3-Rutinoside | C_27_H_30_ O_15_ | Flavonoids | Sophorae Flos |
| 49 | Unknown | C_46_ H_36_O_8_ N_2_ | Unknown | Lonicerae Japonicae Flos |
| 50 | Jionoside B1 | C_37_H_50_O_20_ | Phenylethanoid glycosides | Rehmanniae Radix |
| 51 | Narcissoside | C_28_ H_32_O_16_ | Flavonoids | Rehmanniae Radix |
| 52 | Isochlorogenic acid A | C_25_H_24_O_12_ | Organic acids | Lonicerae Japonicae Flos |
| 53 | Unknown | C_21_H_34_O_11_ | Unknown | Lonicerae Japonicae Flos |
| 54 | Isoacteoside | C_29_H_36_O_15_ | Phenylethanoid glycosides | Sophorae Flos |
| 55 | Icariside F2 | C_18_H_26_O_10_ | Flavonoids | Lonicerae Japonicae Flos |
| 56 | 4'-O-beta-D-Glucosyl-5-O-methylvisamminol | C_22_H_28_O_10_ | Chromones | Saposhnikoviae Radix |
| 57 | Isochlorogenic acid C | C_25_H_24_O_12_ | Organic acids | Lonicerae Japonicae Flos |
| 58 | Centauroside or isomer | C_34_H_46_O_19_ | Terpenoids | Lonicerae Japonicae Flos |
| 59 | 4-(3,5-dihydroxy-7-(4-hydroxyphenyl)heptyl)benzene-1,2-diol | C_19_H_24_O_5_ | Diarylheptanoids | Curcumae Rhizoma |
| 60 | Unknown | C_20_H_26_O_6_ | Unknown | Rehmanniae Radix |
| 61 | 4-(3,5-dihydroxy-7-(4-hydroxyphenyl)heptyl)benzene-1,2-diol isomer | C_19_H_24_O_5_ | Diarylheptanoids | Curcumae Rhizoma |
| 62 | Centauroside or isomer | C_34_H_46_O_19_ | Terpenoids | Lonicerae Japonicae Flos |
| 63 | 6-O-E-Feruloylajugol | C_25_ H_32_O_12_ | Terpenoids | Rehmanniae Radix |
| 64 | Unknown | C_27_H_30_O_14_ | Unknown | Sophorae Flos |
| 65 | Martynoside/ Isomartyside | C_31_H_40_O_15_ | Phenylethanoid glycosides | Rehmanniae Radix |
| 66 | 1,7-bis(4-hydroxyphenyl)heptane-3,5-diol | C_19_H_24_O_4_ | Diarylheptanoids | Curcumae Rhizoma |
| 67 | Quercetin | C_15_H_10_O_7_ | Flavonoids | Sophorae Flos |
| 68 | Sec-O-glucosylhamaudol | C_21_ H_26_O_10_ | Chromones | Saposhnikoviae Radix |
| 69 | 1,7-bis(3,4-dihydroxyphenyl)-5-hydroxyheptan-3-yl acetate | C_21_H_26_O_7_ | Diarylheptanoids | Curcumae Rhizoma |
| 70 | (E)-4-(3-hydroxy-7-(4-hydroxyphenyl)hept-6-enyl)benzene-1,2-diol | C_19_ H_22_O_4_ | Diarylheptanoids | Curcumae Rhizoma |
| 71 | 1-(3,4-dihydroxyphenyl)-5-hydroxy-7-(4-hydroxyphenyl)heptan-3-yl acetate | C_21_H_26_O_6_ | Diarylheptanoids | Curcumae Rhizoma |
| 72 | Isorhamnetin | C_16_H_12_O_7_ | Flavonoids | Curcumae Rhizoma |
| 73 | 1,7-bis(3,4-dihydroxyphenyl)heptane-3,5-diyl diacetate | C_23_H_28_O_8_ | Diarylheptanoids | Curcumae Rhizoma |
| 74 | (3S)-/(3R)-1,7-Bis(4-hydroxyphenyl)-(6E)-6-hepten-3-ol | C_19_H_22_O_3_ | Diarylheptanoids | Curcumae Rhizoma |
| 75 | (3S)-and (3R)-3-acetoxy-1-(3,4-dihydroxyphenyl)-7-(4-hydroxy-phenyl)heptanes | C_21_H_26_O_5_ | Diarylheptanoids | Curcumae Rhizoma |
| 76 | (E)-1,7-bis(4-hydroxyphenyl)hept-6-en-3-one | C_19_H_20_O_3_ | Diarylheptanoids | Curcumae Rhizoma |
| 77 | 1,7-bis(4-hydroxyphenyl)hepta-4E-6E-dien-3-one | C_19_H_18_O_3_ | Diarylheptanoids | Curcumae Rhizoma |
| 78 | 1-(3,4-dihydroxyphenyl)-7-(4-hydroxyphenyl)heptane-3,5-diyl diacetate | C_23_H_28_O_7_ | Diarylheptanoids | Curcumae Rhizoma |
| 79 | Curcumenol | C_15_H_22_O_2_ | Terpenoids | Curcumae Rhizoma |
| 80 | Atractylodin | C_13_H_11_O | Polyacetylene | Atractylodis Rhizoma |
| 81 | Atractylenolide III | C_15_ H_20_O_3_ | Terpenoids | Atractylodis Macrocephalae Rhizoma |
| 82 | Zederone | C_15_H_18_O_3_ | Terpenoids | Curcumae Rhizoma |
| 83 | Azukisaponin I | C_42_H_68_O_13_ | Terpenoids | Sophorae Flos |
| 84 | Atractylenolide II | C_15_H_20_O_2_ | Terpenoids | Atractylodis Macrocephalae Rhizom, Atractylodis Rhizoma |
| 85 | Atractylone | C_15_H_20_O | Terpenoids | Atractylodis Macrocephalae Rhizom |
| 86 | Curzerenone/Furanodienone | C_15_H_18_O_2_ | Terpenoids | Curcumae Rhizoma |
| 87 | Unknown | C_23_H_23_O_5_ | Unknown | Atractylodis Macrocephalae Rhizom |
| 88 | Atractylodinol | C_13_H_10_O_2_ | Terpenoids | Atractylodis Rhizoma |
| 89 | Atractylenolide I | C_15_H_18_O_2_ | Terpenoids | Atractylodis Macrocephalae Rhizom, Atractylodis Rhizoma |
| 90 | Germacrone | C_15_H_22_O | Terpenoids | Curcumae Rhizoma |
| 91 | Dehydrocitrene | C_15_H_22_ | Terpenoids | Atractylodis Macrocephalae Rhizom |

**Table S2**

The sequences of siRNAs/shRNAs used in this study.

| Name | Sequences |
| --- | --- |
| si-YAP/shYAP | F：5’-CUGCCACCAAGCUAGAUAATT-3’  R：5’-UUAUCUAGCUUGGUGGCAGTT-3’ |
| Negative control | F：5’-GCGACGAUCUGCCUAAGAUTT-3’  R：5’-AUCUUAGGCAGAUCGUCGCTT-3’ |

siRNA: short interfering RNAs; shRNAs: short hairpin RNAs.

**Table S3**

The dermatitis score and ear thickness of the mice.

| Group | dermatitis score | | | ear thickness（mm） | | |
| --- | --- | --- | --- | --- | --- | --- |
|  | male | | female | male | female | |
| Ctrl | 0 | 0 | | 0.150±0.012 | | 0153±0.012 |
| AD | 4.000±1.000 | 3.667±1.528 | | 0.207±0.021 | | 0.210±0.010 |
| AD+0.5 AESS | 3.667±0.577 | 4.333±1.155 | | 0.200±0.010 | | 0.203±0.006 |
| AD+AESS | 2.000±1.000 | 1.667±0.577 | | 0.183±0.006 | | 0.177±0.006 |
| AD+2 AESS | 1.667±0.577 | 1.000±0.000 | | 0.173±0.006 | | 0.163±0.006 |
| AD+sh-YAP+AESS | 4.333±1.155 | 4.000±1.000 | | 0.237±0.015 | | 0.237±0.116 |
| AD+DEX | 1.333±0.577 | 1.000±0.000 | | 0.153±0.006 | | 0.170±0.010 |

Ctrl: control mice; AD, atopic dermatitis model mice; AD+0.5 AESS: mice intragastrically administered of 1/2 of the standard dose of AESS formula; AD + AESS: mice intragastrically administered the standard dose of AESS formula; AD +2 AESS: mice intragastrically administered of 2 times of the standard dose of AESS formula; AD + sh-YAP + AESS: mice of the AD + AESS group injected with shRNA-YAP lentivirus; AD + DEX: mice intragastrically administered 3 mg/kg dexamethasone.

**Table S4**

Trans Epidermal Water Loss (TEWL) of the mice (g/m^2^hr).

| Group | Week 1 | | Week 2 | | Week 3 | |
| --- | --- | --- | --- | --- | --- | --- |
|  | male | female | male | female | male | female |
| Ctrl | 6.222±0.509 | 6.556±0.193 | 6.889±0.385 | 6.889±0.385 | 6.889±0.385 | 6.666±0.577 |
| AD | 6.889±1.071 | 6.556±0.839 | 10.778±0.962 | 11.556±1.836 | 17.444±1.644 | 16.778±1.503 |
| AD+0.5 AESS | 7.222±1.018 | 6.667±1.334 | 9.778±1.262 | 9.111±0.770 | 15.111±0.509 | 14.555±0.694 |
| AD+AESS | 6.556±0.839 | 6.889±0.694 | 8.667±0.577 | 7.222±0.509 | 10.556±0.839 | 11.000±2.309 |
| AD+2 AESS | 6.111±0.694 | 6.556±0.770 | 6.333±0.882 | 6.111±0.509 | 8.000±0.333 | 8.222±1.262 |
| AD+sh-YAP  +AESS | 7.111±0.385 | 7.111±0.694 | 12.556±0.839 | 12.000±2.082 | 18.778±0.509 | 18.778±0.509 |
| AD+DEX | 7.667±0.334 | 7.778±0.962 | 11.778±1.018 | 11.778±1.575 | 18.111±0.509 | 17.667±1.667 |

Ctrl: control mice; AD, atopic dermatitis model mice; AD+0.5 AESS: mice intragastrically administered of 1/2 of the standard dose of AESS formula; AD + AESS: mice intragastrically administered the standard dose of AESS formula; AD +2 AESS: mice intragastrically administered of 2 times of the standard dose of AESS formula; AD + sh-YAP + AESS: mice of the AD + AESS group injected with shRNA-YAP lentivirus; AD + DEX: mice intragastrically administered 3 mg/kg dexamethasone.

**Table S5**

Stratum corneum hydration (SCH) of the mice (a.u.).

| Group | Week 1 | | | Week 2 | | | Week 3 | | |
| --- | --- | --- | --- | --- | --- | --- | --- | --- | --- |
|  | male | female | | male | female | | male | female | |
| Ctrl | 20.444±1.836 | | 20.334±0.577 | 20.444±0.193 | | 21.889±1.347 | 20.222±0.509 | | 19.778±0.770 |
| AD | 18.111±1.071 | | 19.222±1.388 | 15.222±0.509 | | 14.889±1.071 | 12.111±0.385 | | 10.111±0.694 |
| AD+0.5 AESS | 19.333±0.882 | | 19.333±1.000 | 15.889±1.678 | | 16.666±1.155 | 12.000±1.528 | | 11.222±0.839 |
| AD+AESS | 19.667±1.000 | | 20.556±0.839 | 19.333±1.000 | | 18.556±0.193 | 16.556±0.839 | | 16.111±0.694 |
| AD+2 AESS | 20.444±1.018 | | 19.667±0.667 | 19.778±0.694 | | 19.889±0.839 | 18.333±0.667 | | 18.666±1.155 |
| AD+sh-YAP  +AESS | 19.334±1.155 | | 19.444±0.839 | 12.000±1.528 | | 12.778±1.895 | 8.889±0.694 | | 8.111±1.071 |
| AD+DEX | 18.556±0.509 | | 18.778±0.509 | 14.222±0.509 | | 14.222±0.694 | 10.444±0.509 | | 10.333±0.882 |

Ctrl: control mice; AD, atopic dermatitis model mice; AD+0.5 AESS: mice intragastrically administered of 1/2 of the standard dose of AESS formula; AD + AESS: mice intragastrically administered the standard dose of AESS formula; AD +2 AESS: mice intragastrically administered of 2 times of the standard dose of AESS formula; AD + sh-YAP + AESS: mice of the AD + AESS group injected with shRNA-YAP lentivirus; AD + DEX: mice intragastrically administered 3 mg/kg dexamethasone.

**Supplementary Figures**

**Fig. S1.** The effect of gu-ben-hua-shi (AESS) on inflammatory disorder in atopic dermatitis (AD) patients. (A) Flow cytometry results from the clinical patients before/after AESS treatment. (B) Scatter plot of IL-4, IL-17A, Th1/Th2 and Th17/Treg of the clinical patients before/after AESS treatment. **P*<0.05 *v.s.* Baseline

**Fig. S2.** The effect of gu-ben-hua-shi (AESS) on autophagy in atopic dermatitis (AD) mice models. (A) Immunofluorescence analysis of YAP subcellular localization. (B) LC3 positive cells in mice skin lesions from immunofluorescence (left: 400×; middle: 600×; right:1000×, red arrows indicate LC3 fluorescent points).

**Fig. S3.** The effect of gu-ben-hua-shi (AESS) on atopic dermatitis (AD) like inflammatory cell model. (A) Flow cytometry analysis of cell apoptosis rate results. (B) Immunofluorescence analysis of YAP subcellular localization. (C) LC3 positive cells in cells from immunofluorescence (left: 400×; middle: 600×; right:1000×, red arrows indicate LC3 fluorescent points).

**Supplementary Methods**

**Sample size calculation**

Based on previous studies (Ping, 2021), the threshold was set at 70% and the expected value was set at 90%. Under these conditions, α = 0.05, β = 0.2, significance level of 0.05 and 80% power (PASS statistic software, version 15.0, NCSS), the total sample size was 26 patients, and assuming a dropout rate of 20% by considering ineligible subjects, a total of 34 individuals were included in the study.

**Reference**

Ping RY. 2021. An exploratory study on the metabolomics of patients with eczema and the effect of Gubenhuashi Decoction on eczema and metabolomics [phd’s thesis], Guangzhou University of Chinese Medicine.
